# Investigating the Interacting Mechanisms of Precast Intersecting Cracks in Concrete: Insights from the Simulations Based on Discrete Element Method

**DOI:** 10.3390/ma19194097

**Published:** 2026-09-24

**Authors:** Yuexing Yu, Wenxin Li, Zhiwei Sun, Liang Liu, Zhiyong Zhang, Runlin Luo, Xianzheng Zhu, Shuyang Yu

**Affiliations:** 1 The Fifth Geological Brigade of Jiangxi Geological Bureau, Xinyu 338000, China; 2 College of Earth Sciences, Guilin University of Technology, Guilin 541004, China; luorunlin@glut.edu.cn; 3 State Key Laboratory of Disaster Prevention and Ecology Protection in Open-Pit Coal Mines, Shandong University of Science and Technology, Qingdao 266590, China; 4 School of Civil Engineering, Qingdao University of Technology, Qingdao 266520, China; 5 Ying’er Command Center, Dongxin Oil Production Plant Shengli Oilfield, Sinopec, Dongying 257000, China; 6 College of Environmental Science and Engineering, Guilin University of Technology, Guilin 541004, China; 7 East China University of Technology, Nanchang 330000, China; 8 School of Transportation and Civil Engineering, Nantong University, Nantong 226019, Chinayushuyang_hhu@163.com (S.Y.)

**Keywords:** discrete element method, concrete, pre-fabricated intersecting fractures, uniaxial compression, fracture propagation

## Abstract

Underground mines often use shotcrete and reinforced concrete as support. Excavation disturbance, in situ stress, and blasting cause initial fractures to propagate. Intersecting fractures are common. This paper studies how intersecting fracture geometry affects fracture propagation under uniaxial compression. DEM and Particle Flow Code (PFC2D, Particle Flow Code) are used to build mesoscopic models with single-intersecting fractures (30–150°) and double-intersecting fractures with five offset levels: Zero, Minor, Moderate, Substantial, and Maximum. For single-intersecting fractures, smaller angles cause stronger shear slip and more multi-source cracking. Peak stress, ultimate strain, and final crack number (defined as the sum of tensile and shear micro-cracks from parallel-bond failure) increase. Larger angles lead to brittle failure and weaker properties. For double-intersecting fractures, bearing capacity first increases then stabilizes with offset. Zero and Minor offset have lower strength; Moderate, Substantial, and Maximum offset have higher capacity. Crack number increases with offset. Maximum offset has the most cracks but the smallest ultimate strain, while Substantial offset has the largest, with deformation from shear slip and higher brittle failure risk. Double-intersecting fracture specimens have fewer cracks but more concentrated damage and weaker properties than single-fracture specimens. This indicates damage distribution, not crack number, governs degradation. This study reveals how fracture configuration controls concrete response, providing mechanistic references for understanding failure behaviors related to safety-analysis and reinforcement-oriented research.

## 1. Introduction

Underground mining engineering often uses shotcrete and cast-in-place concrete as the main support materials. These materials are used in roadways, stopes, pump rooms, and winch chambers. Excavation unloading, high in situ stress, and cyclic blasting vibration continuously damage concrete supports. These actions form many intersecting primary and secondary cracks inside the support layer. These pre-fabricated intersecting fractures greatly change the internal stress transfer path. They accelerate crack initiation and propagation. They cause progressive failure of the support. This seriously threatens underground mining safety [1,2,3]. Damage during service causes safety threats and economic losses to users. In long-term maintenance, cracks in concrete are inevitable. This is especially true under tension and compression. Therefore, studying the long-term crack resistance of concrete is crucial [4,5].

Current studies mostly focus on the mechanical properties of concrete itself. They often ignore internal defects such as existing fractures. These defects affect crack development. This makes it hard to guide and handle sudden problems during long-term maintenance [6,7]. Therefore, it is necessary to study crack development laws of concrete under uniaxial compression. The effects of pre-fabricated intersecting fracture geometry on crack number, propagation path, and coalescence mode should be analyzed. The coupling effect of tip stress concentration and mesoscopic material heterogeneity should also be studied. This has important theoretical value and practical engineering significance. It helps improve concrete fracture mechanics theory. It helps establish bearing capacity evaluation methods for defective structures. It also helps formulate reinforcement and repair strategies.

Laboratory testing is the most direct way to study crack development in concrete. Mechanical instruments apply loads to prepared concrete specimens. The change in crack number and mechanical indexes during the process is studied. Many scholars have conducted extensive research. For example, Li et al. [8] used uniaxial compression tests. They studied the mechanical properties and constitutive relationship of steel fiber-reinforced geopolymer concrete (SFRGC) under stirrup confinement. Xue et al. [9] used prism specimens with different steel slag fine aggregate contents. They conducted uniaxial compression static and fatigue loading tests. They studied the effects of steel slag fine aggregate content on fatigue life, deformation, and damage evolution of steel slag fine aggregate concrete. Liu et al. [10] used milled steel fiber concrete with different fiber contents. They conducted uniaxial compression tests. They studied the effects of fiber type and milled steel fiber content on compression performance, failure mode, and toughness. Chen et al. [11] used rubber particles and macro synthetic fibers to modify concrete. They conducted uniaxial compression tests combined with acoustic emission monitoring. They studied the effects of the two admixtures on damage evolution, crack development, and spatiotemporal distribution of damage modes. Yang et al. [12] used basalt fiber-reinforced coral aggregate concrete. They conducted uniaxial compression tests. They studied the effects of confining pressure ratio, basalt fiber content, and strength grade on multiaxial mechanical properties and failure modes. Zhou et al. [13] used recycled coarse and fine aggregate concrete. They conducted axial compression tests. They studied the effects of recycled coarse and fine aggregate replacement rate and water–binder ratio on compressive stress–strain behavior and internal damage evolution. Xie et al. [14] used manufactured sand concrete with different stone powder contents. They conducted uniaxial compression tests after high temperature treatment. They studied the effects of stone powder content and high temperature on residual mechanical properties, energy evolution, and damage development.

Laboratory testing is a basic method for studying crack development laws. Its advantage is that it can systematically analyze the influence of a single factor. It can eliminate complex on-site interference. It can also be combined with digital image correlation, acoustic emission, and other measurement techniques. This allows high-precision real-time tracking of the whole failure process. It provides reliable data for theoretical verification. However, laboratory testing also has obvious limitations. The specimen size is limited. It is difficult to reflect the size effect and macroscopic heterogeneity of actual structures. The loading boundary conditions are oversimplified. It cannot truly simulate multi-field coupling environments such as in situ stress, seepage, and temperature. In addition, the results are highly sensitive to sample preparation accuracy and initial defects. There is significant data dispersion. The repeated cost is high. The internal information of the failure process is difficult to observe directly. The field quantities at crack tips still need theoretical inversion.

Theoretical analysis is an important way to study and predict crack development in concrete. It uses mathematical formulas and models. Based on fracture mechanics, damage mechanics, and continuum mechanics, scholars have conducted many model constructions and mathematical derivations. For example, Hua et al. [15] used the mechano-electric response theory of concrete. They established a resistivity mathematical model considering curing age and rockfill ratio. They studied the resistivity evolution mechanism and mechano-electric coupled damage evolution of rockfill concrete under compression. Zhang et al. [16] used continuum damage mechanics and statistical strength theory. They studied the true triaxial stress–strain evolution of foamed concrete under pore collapse and skeleton compaction mechanisms. Shen et al. [17] used closed-form beam theory and Menetrey–Willam plastic theory. They studied the mechanical behavior of polypropylene fiber concrete filled steel tube joints under lateral load. They revealed the stress redistribution mechanism of fibers after crack development. Ren et al. [18] used continuum damage mechanics, random mesoscopic generation method, and topological damage definition. They studied the fracture evolution mechanism of concrete composites under uniaxial compression. Xu et al. [19] used Weibull distribution random damage theory. They studied the damage evolution law of materials during the whole compression process. Tang et al. [20] used the time–temperature equivalence principle. They studied the mechanical property degradation of acidic aggregate hydraulic asphalt concrete under long-term water immersion and strain rate coupling. Shang et al. [21] used stress field superposition distribution function and modified softening sleeve theory. They studied the interfacial bond-slip evolution under freeze–thaw cycles and lateral compressive stress coupling.

The advantage of theoretical analysis is that it can establish mechanical criteria for crack initiation, propagation, and coalescence through rigorous mathematical derivation. It can explain the internal relations among stress field, strain field, and energy dissipation from the physical essence. Based on the theoretical framework of elasticity, fracture mechanics, and damage mechanics, researchers can obtain analytical expressions of relevant parameters. This provides a basis for quantitative evaluation of fracture stability. At the same time, theoretical models are universal. They can be applied to regular predictions under different materials and boundary conditions. They are not restricted by test equipment and site conditions.

However, theoretical analysis also has obvious limitations. The heterogeneity, nonlinearity, and anisotropy of real concrete are extremely complex. Strict mathematical analysis often cannot cover all influencing factors. Simplified assumptions and idealized boundaries must be introduced. This may cause deviations between theoretical results and engineering practice. For complex fracture shapes or multi-crack interaction problems, analytical solutions face great mathematical difficulties. They can often only handle a few simplified typical cases. In addition, the validity of theoretical models highly depends on the reasonable selection of constitutive relations and failure criteria. Theoretical derivation without experimental verification cannot independently guide engineering practice.

Numerical simulation is an analysis method based on computer technology and mathematical physical models. It digitally reproduces and predicts actual physical processes through numerical calculation. In the study of concrete under uniaxial compression, this method can fully reproduce the whole loading process. It can track the mesoscopic crack propagation path. It can reveal the damage and failure mechanism. Therefore, it is an indispensable core part of concrete mechanical property research. Compared with laboratory testing, numerical simulation is not restricted by test conditions. It can clearly show mesoscopic processes such as crack initiation, propagation, and coalescence that are difficult to observe directly. It efficiently supports mechanical response analysis and model validation. Therefore, scholars have conducted many simulation calculations and model improvements around deformation characteristics, crack evolution laws, and overall failure modes of concrete under uniaxial compression. They mainly use the finite element method (FEM) and the discrete element method (DEM). Among them, FEM can be based on the continuum mechanics framework. It embeds damage or fracture constitutive models. It can efficiently simulate the whole process of crack initiation, propagation, and coalescence under uniaxial compression. It is also convenient for parameter analysis and model validation. For example, Wu et al. [22] used FEM. They studied the stress transfer mechanism and in situ stress inversion calculation law of concrete under uniaxial compression. José Álvarez-Pérez et al. [23] used FEM. They studied the compressive strength and elastic modulus of ungrouted hollow concrete masonry under gravity. Xu et al. [24] used FEM. They studied the axial compression performance and working mechanism of unequal-leg stiffened L-shaped concrete-filled steel tube short columns under different leg length ratios. Jelena Nikolić et al. [25] used FEM. They studied the axial compression behavior of recycled aggregate concrete-filled steel tube columns. Zhang et al. [26] used FEM. They studied the axial compression performance of a new pre-fabricated CFDST column–column grouted connection. They also studied component interaction, internal force distribution, and load transfer mechanism. Chen et al. [27] used FEM. They studied the advantage of FEM in revealing the higher damage degree and energy dissipation cracking mechanism of concrete under compression. Zhu et al. [28] used FEM. They studied the uniaxial compression performance of recycled sand concrete (RSC) under different recycled sand contents and strength grades.

DEM can directly simulate discontinuous deformation, large displacement, fracture, and contact interaction of granular materials. It does not need a preset failure path. It is especially suitable for dynamic analysis of rock, soil, granular materials, and crushing processes. It provides a reliable way to study crack development of concrete under uniaxial compression. For example, Marek Krzaczek et al. [29] used DEM. They studied the effects of moisture content, strain rate, fluid saturation, and fluid viscosity on dynamic strength and fracture process of low-porosity concrete under uniaxial compression. Zhang et al. [30] used DEM. They studied the mechanical behavior, crack propagation characteristics, and energy evolution mechanism of hybrid fiber rubber concrete (HFRC). Cai et al. [31] used DEM. They studied the damage evolution law of concrete under uniaxial compression with different ITZ strengths. Jiang et al. [32] used DEM. They studied the mechanical behavior of reinforced concrete beams under four-point bending. Wang et al. [33] used DEM. They studied the macroscopic mechanical properties and mesoscopic failure modes of large-size recycled coarse aggregate self-compacting concrete (LRCASCC) under cube uniaxial compression, splitting tension, and prism axial compression. Li et al. [34] used DEM. They studied the effect of aggregate strength on dynamic splitting failure behavior, force chain network distribution, anisotropy tensor, and overall fracture resistance of concrete. Zhao et al. [35] used DEM. They studied the mechanical properties of carbonated recycled aggregate concrete under different carbonation degrees, replacement rates, and old mortar contents. These properties include elastic modulus, compressive strength, and stress distribution.

From the above studies, DEM has significant advantages in studying concrete mechanical properties and crack development. DEM does not need a preset crack path. It can naturally simulate the whole process of crack initiation, propagation, and coalescence in aggregates, mortar, and the interfacial transition zone (ITZ). It truly reflects the discontinuous failure characteristics inside the material. DEM can also simultaneously track the evolution of force chain networks and energy dissipation processes. It establishes the relationship between microscopic contact failure and macroscopic stress–strain response. This provides mechanistic support for explaining the size effect and strain rate effect of concrete compression failure. Therefore, considering these advantages of DEM, this paper chooses DEM to study the crack development mode of concrete under uniaxial compression. Physically informed microstructural DEM models have proven powerful for revealing how internal mineralogical and mesostructural features control macroscopic compressive mechanical responses of brittle geomaterials [36,37]. Combined with image-based mineral feature analysis, even machine-learning tools can help predict uniaxial compressive strength from mesoscopic characteristics [38].

## 2. Principles of Pfc

### Principle of Crack Simulation in Pfc

In Particle Flow Code (PFC2D 5.0), the simulation of crack development is based on the discrete element method. The research object is discretized into a finite number of rigid particles. The particles interact through contact constitutive models. Figure 1 shows the PFC parallel-bond model. The parallel-bond model is commonly used to represent breakable solids. This model introduces a bond disk at each particle contact. The bond disk can transmit force and moment. Its mechanical behavior is controlled by mesoscopic parameters such as normal stiffness, shear stiffness, tensile strength, and cohesion.

All numerical simulations in this work were performed using Particle Flow Code 2D (PFC2D 5.0) Version 5.00.25, developed by Itasca Consulting Group, Inc., Minneapolis, MN, USA.

Crack initiation is determined by the stress state inside the bond disk. Under external load, relative displacement occurs between particles. Normal tensile stress and tangential shear stress develop inside the bond disk. According to the maximum stress criterion, the bond breaks when the tensile stress at the bond disk edge reaches the tensile strength or the shear stress reaches the shear strength. After bond failure, a micro-crack is recorded at that contact position. In this study, crack number denotes the total sum of tensile micro-cracks and shear micro-cracks triggered by parallel-bond breakage at particle contacts. Macro-cracks formed through the coalescence of multiple micro-cracks are not counted as independent cracks. The load-bearing capacity at that location disappears.

Crack propagation is related to stress redistribution after bond failure. The breakage of a single bond transfers its original load to nearby contacts. This changes the local stress field. If the stress at an adjacent bond exceeds its strength threshold after redistribution, a new cracking event occurs. As the load continues to increase, the number of micro-cracks increases. The crack positions tend to align with the direction of maximum stress. The formation order and spatial distribution of micro-cracks depend on the real-time evolution of contact stress between particles. Each cracking event is determined by the current stress field.

Crack evolution appears as the clustering and connection of micro-cracks in space. When the micro-crack density in a local region reaches a certain level, the particle bridges between adjacent micro-cracks break. Cracks coalesce to form macroscopic cracks. Based on the loading characteristics of the bond disk at failure micro-cracks can be divided into tensile type (dominated by normal stress) and shear type (dominated by shear stress). The ratio of the two types changes dynamically during loading. The final crack path, shape, and failure surface are completely determined by the accumulated results of contact cracking events at the particle level. They do not depend on preset macroscopic fracture criteria or path assumptions. During the whole simulation, crack initiation, propagation, and coalescence follow a unified contact fracture criterion. Each cracking event depends only on the comparison between the stress state of each bond disk and its corresponding strength at the current time step. The unique mechanism of PFC can fully show the physical process from mesoscopic damage accumulation to macroscopic fracture formation.

## 3. Numerical Model and Parameters

### 3.1. Setting of Mesoscopic Parameters

In the PFC numerical model, the mechanical parameters of concrete are set mainly based on relevant literature on mesoscopic mechanical simulation of concrete. The settings also incorporate our team’s previous experience in using PFC2D for concrete crack research. The selected parameters include particle contact elastic modulus (Emod), parallel-bond modulus (Pb_emod), parallel-bond tensile strength (Pb_ten), parallel-bond cohesion (Pb_coh), parallel-bond friction angle (Pb_PFA), normal-to-shear stiffness ratio (Kratio), and damping coefficient (damp). The values of each parameter are taken from the common range used in similar PFC studies. They are also initially estimated using empirical formulas.

The mesoscopic parameters summarized in Table 1 are quoted from our previous publication “Numerical Simulation of Crack Propagation in Concrete with Prefabricated Array Fractures Based on the Discrete Element Method” [39]. In that earlier study, substantial trial calculations and parameter screening work were carried out for PFC2D concrete models. Multiple groups of tentative parameter combinations were tested, and inappropriate values that caused excessively high or low stiffness, unrealistic strength or abnormal crack-growth patterns were eliminated. The final parameter set reported in that reference could reproduce the basic mechanical characteristics and fracture behaviors of concrete under compressive loading, which provides a solid basis for parameter adoption in this work. By directly using this validated parameter set, we can guarantee the rationality of parameter settings in our current simulation. Meanwhile, simple explanations are provided to clarify the intrinsic connection between these mesoscopic input variables and the macroscopic mechanical responses of synthetic concrete, so readers can understand how particle-scale parameters govern the overall deformation and failure performance of numerical specimens.

To further verify the rationality of the adopted parameter set, additional uniaxial-compression simulations of intact concrete specimens without pre-fabricated fractures were performed. The numerical results were calibrated against experimental data reported by Ren et al. [40], as illustrated in Figure 2. Good consistency between simulation and test results is achieved in terms of elastic modulus and peak compressive strength. Figure 2b presents the typical failure morphology of the corresponding laboratory-test specimen. It should be pointed out that obvious deviation exists in the post-peak segment. Such discrepancy is mainly caused by the two-dimensional simplification of the PFC2D model. The 2D simulation cannot completely reproduce the real three-dimensional crack propagation and rapid strain localization that occur in laboratory specimens. Therefore, priority is given to matching the elastic stage and peak-strength characteristics, which dominate the pre-failure mechanical responses for the subsequent pre-cracked specimen simulations.

There exists a strong coupling relationship between the mesoscopic parameters of the discrete element model and the macroscopic mechanical responses of concrete. Values of mesoscopic parameters such as bond strength, stiffness ratio and friction coefficient directly affect the elastic modulus and peak compressive strength, as well as the crack initiation and propagation characteristics of the model. Accordingly, absolute quantitative results obtained from numerical calculations in this paper, including peak stress and crack number, cannot be directly equivalent to the measured results of real concrete specimens.

Nevertheless, this study performs comparative parametric analysis. All numerical examples share an identical set of mesoscopic parameters, and only the angle and offset of pre-existing intersecting fractures are varied among different cases. Under fully consistent parameter settings, the relative variation trends of mechanical responses and crack evolution across different working conditions are reliable, which can be used to reveal how fracture geometry governs the failure mechanism of concrete. The simulated crack-initiation locations, wing-crack propagation paths and specimen failure modes are compared against experimental results of pre-cracked concrete from public literature. Good consistency in failure modes is observed, which indirectly demonstrates the rationality of the adopted parameter set.

It should be noted that, constrained by parameter-calibration conditions, the outputs of the present model focus on mechanism analysis, and the relevant absolute indices are not suitable for direct quantitative calculation in practical engineering. For real-world engineering applications, mesoscopic parameter calibration should be further carried out combined with laboratory tests on concrete of the corresponding mix proportion.

### 3.2. Computational Model and Specimen Dimensions

In PFC modeling, control code is written. First, the outer boundary of the model is defined as a square area. Its width and height are both set to 0.06 m. The detailed geometry and boundary conditions are shown in Figure 3. The geometric definitions of pre-fabricated intersecting fractures, including fracture intersection angle *α*, fracture trace length *L*, vertical offset *d*1 and horizontal offset *d*2, are illustrated in Figure 4. All pre-fabricated fractures in this study are internal embedded cracks confined inside the numerical specimen and do not extend to specimen boundaries; they are neither superficial surface cracks nor through-penetrating cracks crossing specimen boundaries. Since fractures are generated by removing particles within DXF-defined geometric regions, there is no artificially assigned crack-tip radius; the tip geometry is naturally controlled by the size of surrounding discrete particles. The fracture trace length *L* remains constant for all simulation cases. For double-intersecting fracture specimens, five offset levels (Zero, Minor, Moderate, Substantial, and Maximum) are realized by adjusting vertical offset *d*1 and horizontal offset *d*2. Crack length is not treated as a variable in the present parametric analysis, and its influence is recommended for future investigation.

Then, within the model area, a random generation algorithm is used. It is based on a preset gradation curve. Eight groups of spherical elements with different particle size ranges are generated. They represent the multiphase composition inside concrete. The first seven groups correspond to coarse aggregate particles of different size levels. Their particle size ranges follow a certain gradation. They simulate coarse and fine aggregate components such as crushed stone or pebbles in real concrete. The eighth group consists of fine cement mortar particles. They represent the matrix material filling the gaps between aggregates.

Each group of particles is placed within the square area according to the set gradation ratio and random positions. This ensures uniform spatial distribution and statistical representativeness. Together they form a numerical model that reflects the mesoscopic structural characteristics of concrete. This generation method balances computational efficiency and mesoscopic structural realism.

The 0.06 m × 0.06 m square numerical specimen is generated with a fixed random seed of 10001 to enhance reproducibility. Eight particle bins are defined for mesostructure reconstruction. Bins 1–7 correspond to aggregate particles with different radius ranges: 0.0011–0.0013 m, 0.0012–0.0018 m, 0.0004–0.0015 m, 0.0016–0.0019 m, 0.0015–0.0022 m, 0.0017–0.0021 m, and 0.0023–0.0027 m, each with a volume fraction of 0.06. Bin 8 represents the cement-mortar matrix with particle radii ranging from 0.00015 m to 0.00025 m and a volume fraction of 0.58. The initial porosity of the particle assembly is 0.06, and particle density is assigned as 2600 kg/m^3^. Pre-fabricated intersecting fractures are realized by importing DXF geometry files and deleting particles falling within the geometric fracture region. For single-intersecting fracture specimens, a pair of intersecting fractures creates four included angles. In this study, the geometric parameter (30°, 60°, 90°, 120°, 150°) corresponds to the upper-side internal included angle formed by the two intersecting fracture segments, which serves as the main variable for parametric analysis under uniaxial compressive loading.

Regarding boundary conditions, rigid walls are arranged at the top and bottom boundaries of the specimen, whereas the left and right boundaries are free without wall confinement. Uniaxial quasi-static compressive loading is performed by applying a constant downward velocity to the top wall while keeping the bottom wall fixed. The particle assembly achieves initial mechanical equilibrium under the unbalanced-force ratio criterion ‘aratio = 1 × 10^−4^’, and automatic time-stepping is activated for all simulation cases.

## 4. Simulation Results

### 4.1. Strength Characteristics of Concrete in the Tests

#### 4.1.1. Stress–Strain Curves

The following is an analysis of stress–strain curves for concrete with single and double-intersecting fractures under uniaxial compression.

Based on multiple stress–strain curves from PFC numerical simulation, this paper systematically analyzes the effects of two types of working conditions. The first group is single-intersecting fractures with different angles (30°, 60°, 90°, 120°, 150°). The second group is double-intersecting fractures with different offset levels (Zero offset, Minor offset, Moderate-offset, Substantial offset, Maximum offset). Pre-fabricated intersecting fractures act as initial defects. At the mesoscopic level, they change the internal stress distribution, damage evolution, and load transfer path of concrete. During loading, they promote crack development. Finally, they cause differences in macroscopic bearing capacity. The overall pattern shows the following. For single-intersecting fractures, a smaller angle leads to a higher stress value at the same strain. For double-intersecting fractures, a larger offset leads to a higher stress value at the same strain. The maximum stress of single-intersecting fracture specimens is higher than that of double-intersecting fracture specimens without fractures. This means single-intersecting fracture specimens have better bearing capacity. The following is the analysis of each condition.

(1) Single-intersecting fractures with different angles (30°, 60°, 90°, 120°, 150°)

Note that the angle values quoted herein represent the upper-side internal included angle of two intersecting fractures as defined in Section 3.2. The stress–strain curves obtained by PFC2D particle flow simulation show similar overall patterns. The stress of all specimens first increases and then decreases with increasing strain. This is shown in Figure 5a. In the early-loading stage, stress increases almost linearly with strain. The specimen is in the elastic stage. After reaching the peak stress, the bearing capacity gradually decreases as strain continues to develop. On this common pattern, the fracture angle has a significant regulating effect on the mechanical properties of the specimens. A smaller fracture angle leads to a larger peak stress and a larger final strain at failure. A larger fracture angle leads to lower peak stress and ultimate strain. The mechanical difference between the smallest angle (30°) and the largest angle (150°) is the most obvious. The peak stress of the 30° specimen is about twice that of the 150° specimen. At the same time, medium and high angle conditions show similar mechanical properties. The peak stress values of 90° and 120° specimens are close. The change in fracture angle directly changes the mesoscopic stress state, crack propagation pattern, and overall deformation and failure characteristics. It is a key factor in regulating specimen strength and deformation behavior.

When the fracture angle is small, the angle between the fracture plane and the axial loading direction is small. Under axial load, shear slip easily occurs at the internal fracture interface. The original fractures can extend stably and progressively. The specimen shows an obvious plastic deformation stage. Under this condition, the particle bond contacts inside concrete break gradually. Stress is released slowly. The specimen can still produce some post-peak deformation. It has a larger ultimate strain. At the same time, the gentle fracture orientation can effectively disperse internal stress. It weakens stress concentration. The specimen can bear higher axial load. Therefore, the peak stress is larger overall.

As the fracture angle gradually increases, the fracture orientation tends to be perpendicular to the loading direction. The shear-slip effect of the fracture plane is significantly suppressed. Under loading, the internal stress concentration becomes more severe. Cracks no longer extend slowly along the fracture interface. Instead, they develop rapidly in a sudden and penetrating manner. After reaching the peak strength, the specimen quickly becomes unstable and fails. The plastic deformation stage is greatly shortened. There is almost no post-peak deformation. The final ultimate strain is obviously reduced. At the same time, severe stress concentration accelerates internal bond failure. It reduces the bearing limit of the specimen. The peak stress decreases with increasing angle. Among them, the internal stress concentration and crack propagation mode of the 90° and 120° specimens are similar. Therefore, their peak stresses show good consistency with small numerical differences.

From the mesoscopic mechanism, the change in fracture angle reconstructs the internal force chain transfer path. Small-angle specimens are dominated by shear energy dissipation and plastic deformation. Their stress distribution is uniform. Their deformation reserve and bearing capacity are more sufficient. The 30° specimen has the optimal fracture spatial configuration. Its bearing performance is much better than large-angle specimens. Its peak stress reaches twice that of the 150° specimen. Large-angle specimens have serious stress-concentration problems. Their brittle failure characteristics are obvious. Their bearing capacity and deformation capacity are greatly weakened. In summary, under PFC2D numerical simulation, the peak strength and ultimate deformation capacity of concrete specimens with single-intersecting fractures continuously decrease with increasing fracture angle. The strength difference between small-angle and large-angle specimens is very obvious.

(2) Double-intersecting fractures with different offset levels (Zero offset, Minor offset, Moderate offset, Substantial offset, Maximum offset)

The stress–strain curves of concrete specimens with double-intersecting fractures under different offset levels obtained by PFC2D particle flow simulation also show basically the same pattern. The stress of all specimens first increases and then decreases with increasing strain. This is shown in Figure 5b. In the early-loading stage, stress and strain show an approximately linear relationship. The specimen is in the elastic stage. As loading continues, stress increases to the peak strength. Then internal damage accumulates. Bearing capacity gradually decreases. Finally, unstable failure occurs. On the common curve pattern, the fracture offset level has a significant regulating effect on the strength and deformation characteristics of double-intersecting fracture specimens.

From the peak stress pattern, the peak stress generally increases with increasing offset level. The peak stress values of specimens with different offset levels show obvious grouping convergence. Among them, the peak stress values of Maximum-offset, Substantial-offset, and Moderate-offset specimens are close. Their mechanical strength levels are similar. The peak stress values of Zero-offset and Minor-offset specimens are close to each other. They are significantly lower than those of Moderate-, Substantial-, and Maximum-offset conditions. This shows that moderately increasing the offset level of double-intersecting fractures can effectively improve the bearing capacity of concrete specimens. But after the offset reaches a medium level, further increasing the offset has limited effect on peak strength. The strength gradually becomes stable.

From the deformation characteristics of ultimate strain, the failure strains of specimens with different offset levels show differentiated distribution patterns. The overall ranking is significant. The Substantial-offset specimen has the largest ultimate strain and the best plastic deformation capacity. The Moderate-offset specimen is next. The Zero-offset and Minor-offset specimens have smaller ultimate strains. The Maximum-offset specimen has the smallest ultimate strain and the most obvious brittle deformation characteristics. This shows that the offset level has a two-way regulating effect on plastic deformation reserve. Moderate offset can improve deformation performance. But excessive offset greatly weakens the ultimate deformation capacity of concrete specimens.

From the mesoscopic mechanism, the offset change in double-intersecting fractures changes the internal crack coalescence path and force chain transfer structure. Under low offset conditions, the fracture connectivity is good. The internal stress concentration is significant. The bearing capacity is low and the deformation performance is limited. Moderate- and Substantial-offset conditions can disrupt the overall coalescence trend of fractures. They optimize internal stress distribution. They delay the batch failure of particle bond contacts. They effectively improve bearing strength and plastic deformation capacity. When the offset reaches the maximum, the internal force structure tends to be regular. The crack propagation rate increases. The plastic deformation stage is compressed. Finally, the ultimate strain is significantly reduced.

(3) Summary

Based on the simulation results of the single-intersecting fracture angle effect and the double-intersecting fracture offset effect, both fracture configurations affect the mechanical properties of concrete specimens. For concrete specimens with single-intersecting fractures, the fracture angle is the main controlling factor. The peak stress and ultimate strain are negatively correlated with the fracture angle. A smaller angle leads to better bearing capacity and deformation performance. The peak stress of the 30° specimen is about twice that of the 150° specimen. The peak stresses of the 90° and 120° specimens show highly similar characteristics. For concrete specimens with double-intersecting fractures, the offset level is the dominant factor. The peak stress generally increases with offset level and shows obvious grouping convergence. The ultimate strain has no monotonic pattern and shows significant interval differences.

#### 4.1.2. Crack Number Evolution Curves

(1) Single-intersecting fractures with different angles (30°, 60°, 90°, 120°, 150°)

Through PFC2D particle flow numerical simulation, we obtained the evolution curves of crack number (sum of tensile and shear micro-cracks induced by parallel-bond breakage) under different fracture angles. These curves are shown in Figure 4a. The curves show that the crack number of each group of single-intersecting fracture concrete specimens follows a typical exponential growth law with loading progress. At the early-loading stage, the specimen is in an elastic stress state. The internal stress distribution is uniform. Fewer particle bonds break. The crack initiation rate is slow. The crack number increases slightly. As the axial load continues to increase, the stress-concentration effect inside the specimen intensifies. The initial fractures gradually develop and propagate. Secondary cracks continue to initiate and coalesce. The crack growth rate accelerates significantly. The overall trend shows rapid exponential growth until macroscopic instability failure occurs.

The fracture angle has a significant controlling effect on the final crack number of the specimen. Overall, the smaller the fracture angle, the more final cracks the specimen generates. Among all simulation cases, the specimen with a 30° small angle has the largest final crack number. The specimen with a 150° large angle has the smallest final crack number. Quantitative results show that the final crack number of the 30° case is about three times that of the 150° case. The difference in meso-damage degree is significant. Meanwhile, as the fracture angle gradually increases, the final crack number of the specimen generally decreases. The damage development degree continuously weakens with increasing angle.

From the perspective of meso-failure mechanism, the small-angle fracture has a small angle with the loading axis. During loading, the fracture interface is prone to shear-slip. The original fracture continues to propagate. Moreover, a large number of secondary tensile and shear cracks are induced at the two ends and around the fracture. The internal damaged area is wider. The number of broken bonds increases significantly. For the large-angle fracture, it approaches the direction perpendicular to loading. The shear slip effect is significantly suppressed. The specimen failure mode is mainly sudden brittle coalescence failure. The crack propagation path is single. Secondary cracks develop less. The internal damage range is limited. The final crack number is significantly reduced.

(2) Double-intersecting fractures with different offset degrees (Zero offset, Minor offset, Moderate offset, Substantial offset, Maximum offset)

Based on PFC2D particle flow numerical simulation, as shown in group Figure 6b, the crack number variation curves of double-intersecting fracture specimens with different offset degrees show that during loading the crack number of each group increases continuously. This reflects that the particle bonds inside concrete keep breaking, and meso-cracks experience the whole process of initiation, propagation, and coalescence. Compared with single-intersecting fracture specimens, the final crack numbers of double-intersecting fracture specimens under all offset cases are generally lower. The meso-damage degree is relatively weaker. This indicates that the fracture structure form has a significant regulating effect on the scale of crack development inside concrete.

In the double-intersecting fracture system, the final crack number generally rises with increasing offset within a certain range, but does not increase monotonically all the time. The Zero-offset specimen generates the fewest cracks. The maximum value of final crack number appears under the Substantial-offset condition, and a slight drop is observed for the Maximum-offset specimen. This indicates that increasing offset within a reasonable range can intensify meso-damage and promote crack initiation and propagation, while excessively large offset will suppress further growth of crack quantity.

From the meso-mechanical mechanism analysis, the Zero-offset double-intersecting fracture structure is relatively regular. The fracture coalescence path is smooth. During loading, a single dominant failure surface is easily formed. The crack propagation form is relatively concentrated. The number of secondary cracks is limited. Therefore, the overall damage scale is the smallest. As the offset degree increases, the spatial overlapping relationship of the two fractures is broken. The internal stress distribution becomes more complex. Stress concentration occurs in multiple areas, inducing a large number of secondary meso-cracks. The crack development range is wider and the number is larger. Under the Substantial-offset condition, the spatial overlapping relation of two intersecting fractures is sufficiently disturbed. Multiple stress-concentration zones are formed, which induce abundant secondary meso-cracks and produce the highest crack count. When offset further increases to the Maximum-offset level, the failure mode transforms into a single oblique penetrating path, restricting the generation of secondary cracks and leading to a slight reduction in total crack number.

(3) Summary

Based on the PFC2D particle flow numerical simulation results, we systematically compare and analyze the crack evolution and meso-damage laws of concrete specimens under single-intersecting fracture and double-intersecting fracture configurations. We can obtain unified and differentiated mechanical response characteristics. The unified aspect is that the crack numbers of both types of fracture specimens increase continuously with loading.

For single-intersecting fracture concrete specimens, the fracture angle is a controlling factor for crack propagation. The crack number is negatively correlated with the fracture angle. The smaller the angle, the more significant the shear-slip effect of the fracture. This produces more secondary cracks. The internal damage range is wider, and the final crack number is larger. The larger the angle, the more the shear effect is suppressed, and fewer secondary cracks develop. Among them, the specimen with a 30° angle has the largest final crack number, while the 150° angle specimen has the smallest.

For double-intersecting fracture concrete specimens, the offset degree affects crack evolution. Compared with single-intersecting fractures, the overall crack numbers of specimens under all offset cases are lower, and the meso-damage degree is weaker. For double-intersecting fracture specimens, crack number generally increases with offset within a certain range rather than following a strict monotonic positive correlation. The Zero-offset case yields the fewest cracks, whereas the maximum crack number occurs under the Substantial-offset condition instead of the Maximum-offset condition. The Zero-offset double-fracture structure is regular. After loading, a single failure surface is easily formed, and secondary crack development is limited. As the offset degree increases, the original overlapping structure of fractures is destroyed. The internal stress distribution becomes complex. Stress concentration occurs in multiple regions. The force chain transmission is disordered. This greatly promotes the initiation and propagation of secondary cracks and intensifies internal damage.

#### 4.1.3. Load–Displacement Curves

(1) Single-intersecting fractures with different angles (30°, 60°, 90°, 120°, 150°)

Based on PFC2D numerical simulation, as shown in Figure 7a, the load–displacement curves of single-intersecting fracture concrete specimens with different fracture angles are obtained. The overall evolution trends of the curves under all cases are basically the same. The load first increases and then decreases with displacement. At the early-loading stage, the load increases approximately linearly with displacement. The specimen is in an elastic stress state. As displacement continues to increase, internal damage accumulates. After the load reaches its peak, it gradually drops. Finally, the specimen fails globally. The fracture angle has a significant regulating effect on both the peak load capacity and the ultimate deformation performance.

From the peak load variation, the maximum load capacity generally decreases with increasing fracture angle. The specimen with a 30° angle has the largest peak load and the best load capacity. The 60° specimen has the second largest peak load. The 90° and 120° specimens have similar peak loads, which are at a medium level. The 150° specimen has the smallest peak load and the weakest load capacity. Quantitative results show that the maximum load of the 30° specimen is 1 to 2 times that of the 150° specimen. The difference in load capacity between small- and large-angle specimens is obvious.

From the deformation characteristics at the ultimate displacement, the deformation capacity of different angle specimens also shows clear grading features. The 30° specimen has the largest ultimate displacement and the highest plastic deformation reserve. The 60° and 90° specimens have similar ultimate displacements, ranking second in deformation capacity. The 120° and 150° specimens have nearly the same ultimate displacement, which is the smallest among all cases, and their overall deformation capacity is the weakest. In general, the smaller the fracture angle, the larger the ultimate load and ultimate displacement the specimen can bear, and the better the overall mechanical performance. As the angle increases, both load capacity and deformation capacity weaken, and brittle failure features become more prominent.

(2) Double-intersecting fractures with different offset degrees (Zero offset, Minor offset, Moderate offset, Substantial offset, Maximum offset)

Based on PFC2D particle flow numerical simulation results, as shown in Figure 7b, the load–displacement curves of double-intersecting fracture concrete specimens with different offset degrees are analyzed. The overall evolution laws of all curves are consistent. They all show the typical feature that load first increases and then decreases with displacement. At the early stage, the specimen is in an elastic state, and the load increases linearly with displacement. As loading displacement continues to increase, internal damage accumulates. After the load peaks, it gradually declines, and finally the specimen fails. The offset degree is a key factor affecting the load capacity and deformation characteristics of double-intersecting fracture concrete specimens.

From the peak load variation, different offset cases show obvious grouping characteristics. The peak loads of the Maximum-, Substantial-, and Moderate-offset cases are close to each other. They are the highest among all cases, with the best load capacity. The Minor- and Zero-offset specimens have similar peak loads, which are significantly lower overall, and their load capacity is relatively weak. Overall, moderately increasing the offset degree can effectively enhance the ultimate load capacity of double-intersecting fracture concrete specimens. After the offset reaches a moderate level, the peak load tends to stabilize and does not increase further.

In terms of deformation characteristics, the ultimate displacement of the specimen is positively correlated with the offset degree. The larger the offset, the larger the ultimate displacement at failure, and the stronger the plastic deformation capacity. Increasing the offset can change the spatial combination of the two fractures, disrupt the coalescence path, delay the brittle failure process, and effectively improve the overall deformation reserve of the concrete specimen.

Compared with single-intersecting fracture specimens, the mechanical performance of double-intersecting fracture specimens is generally weaker. Under the same loading conditions, the peak load that double-intersecting fracture specimens can bear is significantly lower than that of single-intersecting fracture specimens. Also, the ultimate displacement is slightly smaller. This indicates that increasing the number of intersecting fractures further aggravates structural damage defects and reduces the overall load and deformation performance of concrete specimens.

(3) Summary

Based on PFC2D numerical simulation results, the load–displacement curve evolution laws of single- and double-intersecting fracture concrete specimens are basically the same. Both show that load first rises and then falls with displacement. For single-intersecting fracture specimens with different angles, the mechanical performance is significantly negatively correlated with the fracture angle. Among them, the 30° specimen has the largest peak load and ultimate displacement, followed by the 60° specimen. The 90° and 120° specimens have similar peak loads. The 120° and 150° specimens have nearly the same ultimate displacement. The peak load of the 30° specimen can be 1 to 2 times that of the 150° specimen.

For double-intersecting fracture specimens with different offset degrees, the peak load shows clear grouping characteristics. The Moderate-, Substantial-, and Maximum-offset specimens have similar and higher peak loads. The Zero- and Minor-offset specimens have lower peak loads. At the same time, the ultimate displacement gradually increases with increasing offset degree.

#### 4.1.4. Summary and Quantitative Comparison

Based on the stress–strain curves, load–displacement curves and crack-number evolution curves presented above, Table 2 summarizes the key quantitative indices for all simulated specimens, including peak stress, ultimate strain, peak load and final crack number. This subsection carries out a comparative analysis of mechanical performance and mesoscopic damage from a quantitative perspective.

For single intersecting-fracture specimens (A-1–A-5), fracture angle exerts a remarkable control over mechanical and damage indices. The specimen with 30° fracture angle achieves the highest peak stress of 15.6 MPa. Compared with the 150° case, its peak stress increases by more than 60%. Two medium-angle specimens (90° and 120°) share identical peak-stress values, which quantitatively validates their similar deformation and failure behaviors. Overall, peak stress and peak load present a continuous decreasing trend with the increase in fracture angle, whereas ultimate strain and final crack number show non-monotonic variation with slight fluctuations at intermediate angles.

For double-intersecting fracture specimens (B-1–B-5), strength indices exhibit obvious grouping characteristics. The peak stress remains stable at 5.5 MPa for Moderate, Substantial and Maximum-offset conditions, indicating that further increase in offset beyond a certain threshold brings no obvious improvement in bearing capacity. Compared with the Zero-offset specimen, Moderate offset effectively improves load-bearing capacity. Among double-fracture cases, the Substantial-offset specimen possesses the largest ultimate strain, about 30% higher than that under Maximum-offset condition. Appropriate offset can stimulate more extensive meso-crack initiation and propagation.

Comparing single- and double-intersecting fracture configurations: under comparable simulation conditions, double-fracture specimens suffer an average peak-stress reduction above 50% relative to single-fracture specimens. This quantitatively confirms that double-intersecting fractures induce severe stress concentration and localized damage, rather than widespread crack generation, resulting in the overall deterioration of macroscopic mechanical properties.

Combined with the qualitative observations of crack propagation patterns in Section 4.2, the above quantitative comparisons further clarify the regulating effect of fracture geometric parameters on the compressive response of pre-cracked concrete.

### 4.2. Crack Count and Morphological Changes

#### 4.2.1. Single-Intersecting Fractures with Different Angles (30°, 60°, 90°, 120°, 150°)

(1) Single-intersecting fractures with small angles (30°, 60°)

Combined with the crack propagation evolution laws of small-angle (30°, 60°) single-intersecting fracture specimens in Figure 8a,b, the crack development of both types of small-angle specimens shows obvious stage characteristics. At the early-loading stage, cracks mainly initiate around the initial single-intersecting fracture. The total crack number is small and scattered. Only a few micro-cracks extend and propagate. No large-scale crack coalescence path has formed. The specimen damage degree is low. At the middle loading stage, the micro-cracks around the fracture continue to develop and expand outward into the specimen. New cracks begin to appear at the specimen edges. Crack accumulation is obvious. The total crack number increases significantly. The internal damage range continues to expand. At the late-loading stage, a large number of micro-cracks concentrate, initiate, develop, and interconnect in the void areas inside the specimen. Cracks enter a stage of large-scale extension and coalescence. Damage spreads throughout the specimen. Many long cracks gradually penetrate the whole specimen structure. The specimen tends to complete instability failure.

Comparing the final crack patterns and numbers of the two small-angle specimens, under the same loading conditions, the final crack number and propagation range of the 60° specimen (group b) are generally smaller than those of the 30° specimen (group a). This indicates that within the small-angle range, as the fracture angle increases slightly, the crack development degree of the specimen weakens, and the internal damage scale is relatively reduced. This is consistent with the overall mechanical law that a smaller angle causes more severe damage.

(2) Single-intersecting fracture with a medium angle (90°)

As shown in Figure 9, at the early-loading stage, the specimen is in an elastic stress state. The internal stress concentration mainly occurs at the four tips of the initial single-intersecting fracture. Micro-cracks first initiate at the four corners of the fracture and slowly extend outward. At this stage, the total crack number is small. The extension scale is limited. Most cracks are discrete micro-cracks. No continuous crack propagation path has formed. The overall internal damage degree is low.

At the middle loading stage, as the external load continues to apply, the internal stress redistributes. The crack propagation shows significant directional differences. Crack growth in the vertical direction is obviously suppressed. Almost no obvious extension occurs. Crack propagation is mainly horizontal to the left and right. Meanwhile, the cracks at the upper and lower corners of the initial intersecting fracture gradually coalesce and connect. They continuously extend toward the lower-left and upper-right corners of the specimen. The crack distribution range continues to expand. The internal damaged area gradually concentrates.

At the late-loading stage, the specimen enters a rapid damage development phase. The number of internal cracks increases significantly. The existing main cracks continue to lengthen and widen. At the same time, a small number of new cracks begin to develop toward the upper-left and lower-right corners. Cracks in different directions intersect and overlap with each other. They form a cross-type crack distribution pattern inside the specimen. The damaged area spreads throughout. A penetrating fracture structure gradually forms. Finally, the specimen experiences overall instability failure. Compared with small-angle specimens, the medium-angle specimen shows stronger directional crack propagation. The number of secondary cracks is smaller. The overall damage degree is relatively weaker. It exhibits more obvious localized failure characteristics.

(3) Single-intersecting fractures with large angles (120°, 150°)

Combined with the simulation results of large-angle single-intersecting fracture concrete specimens in Figure 10a,b, the crack initiation and propagation processes of large-angle fracture specimens also show obvious stage characteristics. Moreover, the crack development direction has strong regularity.

At the early-loading stage, the specimen is in an elastic stress state. The internal stress concentration mainly occurs at the four tips of the single-intersecting fracture. Micro-cracks first initiate at the four corners of the fracture. They slowly extend outward from the tips. At this stage, the crack development degree is low. The crack number is small and the distribution is scattered. No obvious long cracks form. The specimen remains intact overall. The internal damage scale is very small.

At the middle loading stage, as the axial load continues to increase, the internal stress redistributes. The crack propagation direction gradually becomes stable. It shows an obvious horizontal development feature. At this stage, vertical crack growth basically stops. New cracks and existing cracks mainly extend toward the left and right sides of the specimen. The crack distribution range gradually spreads to the lateral areas. The local damage degree continues to increase. However, no penetrating failure surface has yet formed.

At the late-loading stage, the internal damage enters a rapid development phase. The crack number increases significantly. A large number of cracks are concentrated on the left and right sides of the specimen. As loading continues, the horizontal cracks continuously extend, overlap, and coalesce. Finally, a continuous penetrating main crack forms on the left side of the specimen. This main crack dominates the overall failure mode. Overall, the crack propagation path of large-angle single-intersecting fracture specimens is relatively simple. Fewer secondary cracks develop. The failure is mainly unilateral penetrating failure. The brittle failure feature is obvious. The overall internal damage degree is much lower than that of small-angle fracture specimens.

#### 4.2.2. Double-Intersecting Fractures with Different Offset Degrees (Zero Offset, Minor Offset, Moderate Offset, Substantial Offset, Maximum Offset)

(1) Double-intersecting fractures with small offsets (Zero offset, Minor offset)

As shown in Figure 11a,b, the crack propagation processes of Zero-offset and Minor-offset double-intersecting fracture specimens have stage characteristics. The crack initiation locations, propagation paths, and distribution ranges are highly similar. They are also different from the damage development mode of single-intersecting fracture specimens.

At the early-loading stage, the internal stress concentration mainly occurs at the left intersecting fracture. Initial micro-cracks preferentially initiate from the left fracture and slowly extend outward. As loading continues, the cracks gradually extend from the left fracture toward the middle area of the specimen. They continue to slowly develop toward the right intersecting fracture. At this stage, the total crack number is small. The extension speed is slow. Only a few scattered micro-cracks form. The overall damage degree is low.

At the middle loading stage, under continuous loading, the internal stress further redistributes. The middle area between the two fractures becomes the core area of stress concentration. Therefore, the crack number between the two groups of fractures increases significantly. Cracks continuously overlap and develop. The damaged area continues to concentrate in the middle of the specimen. At the same time, a few new micro-cracks appear at the edges. The overall crack distribution range further expands. The internal damage degree continues to increase. However, no penetrating failure surface has yet formed.

At the late-loading stage, the damage characteristics of the specimen tend to stabilize. Cracks are highly concentrated in the middle area between the two intersecting fractures. The main crack gradually penetrates the double-fracture structure, forming a central dominant failure surface. Compared with single-intersecting fracture specimens, the total crack number of small-offset double-intersecting fracture specimens is significantly smaller. Moreover, cracks are only concentrated in the middle area. Almost no cracks initiate or propagate around the specimen edges.

(2) Double-intersecting fractures with moderate offset (Moderate offset)

As shown in Figure 12, the crack initiation and propagation processes of Moderate-offset double-intersecting fracture concrete specimens also show stage characteristics. Compared with Zero-offset and Minor-offset specimens, the crack development path and coalescence mode have changed. The spatial damage distribution is more balanced.

At the early-loading stage, the stress-concentration effect mainly acts on the left intersecting fracture. Micro-cracks preferentially initiate around the left fracture and develop slowly. At this time, the stress response of the right intersecting fracture is weaker. Only a few scattered micro-cracks appear near the fracture. The total crack number is small. The distribution is scattered. There is no obvious propagation trend. The specimen is in a mild damage state. The structural integrity is good.

At the middle loading stage, as the axial load continues to increase, the internal stress redistribution is completed. Both the left and right intersecting fractures enter a rapid damage development phase. The micro-cracks around the two fractures continue to extend and overlap. They gradually connect toward the middle area of the specimen. They slowly form a main crack that runs through the specimen in the vertical direction. At this stage, the main crack initially takes shape. The crack coalescence trend is obvious. The internal damage changes from unilateral concentration to overall coalescence development. The structural integrity of the specimen begins to be damaged.

At the late-loading stage, based on the vertical penetrating main crack formed in the middle stage, the cracks further expand in all directions. The width and development range of the main crack continue to increase. The overall connectivity of the crack is greatly enhanced. It becomes the core structure that dominates specimen failure. Compared with Minor-offset specimens, the Moderate-offset double-intersecting fracture specimen has a wider crack propagation range and a higher coalescence degree. The internal damage is more significant. The failure mode changes from local concentrated damage to overall coalescence damage. The spatial structure change caused by the fracture offset significantly changes the meso-damage evolution mechanism of the specimen.

(3) Double-intersecting fractures with large offsets (Substantial offset, Maximum offset)

As shown in Figure 13, the crack initiation and propagation processes of large-offset double-intersecting fracture concrete specimens also show typical stage characteristics. Compared with Zero-offset, Minor-offset, and Moderate-offset specimens, the crack development pattern is clearer and more regular. The crack propagation path has clear directional features. The overall meso-damage mode is significantly different.

At the early-loading stage, due to the large-offset spatial structure, the stress concentration mainly occurs in the upper-left intersecting fracture area. Micro-cracks preferentially initiate around this fracture and slowly extend. At this time, the lower-right intersecting fracture is less stressed. Only a few scattered micro-cracks appear. The overall crack distribution is sparse. The development degree is very low. There is no obvious propagation trend. The overall structure remains intact. The initial damage scale is small.

At the middle loading stage, as the axial load continues to apply, the internal stress redistributes. The micro-cracks around the upper-left and lower-right intersecting fractures continue to develop and extend toward each other. The cracks on both sides gradually overlap and connect. They form a clear crack propagation path along an oblique trajectory. A diagonal main crack connecting the upper-left and lower-right intersecting fractures is preliminarily formed. This is different from the vertical penetrating crack in the Moderate-offset case. The damage development direction is more definite. The crack pattern is regular and orderly.

At the late-loading stage, based on the diagonal main crack formed in the middle stage, the cracks further extend, propagate, and coalesce. The formation and connectivity of the main crack are greatly enhanced. The overall crack network becomes clearer and more complete. It becomes the core structure that controls specimen failure. Compared with small- and moderate-offset cases, the crack propagation path of the large-offset specimen is more regular. No messy secondary cracks appear. The oblique penetrating damage feature is extremely significant. The spatial configuration of the large-offset fractures effectively dominates the directional development of cracks. It makes the damage mode more single and the crack network clearer. The meso-failure mechanism is different from that of low-offset double-intersecting fracture specimens.

## 5. Discussion

### 5.1. Effect of Single-Intersecting Fractures with Different Angles on Crack Development

With a small angle, shear slip induces a large number of secondary tensile and shear cracks. There are many initiation points. Propagation paths are rich. The damage range is wide. With a large angle, shear slip is suppressed. Failure is brittle coalescence. The path is single. Secondary cracks are few. The damage range is limited. The difference in crack number essentially reflects a transition in energy dissipation mechanism. Small-angle specimens dissipate energy through extensive crack initiation and propagation, showing obvious ductility. Large-angle specimens release energy in a concentrated manner, showing prominent brittleness.

Crack propagation observations show that as the angle increases from small to large, the propagation mode changes from multi-source progressive to single coalescence. Under small-angle conditions, cracks are scattered around the fracture at the early stage. At the middle stage, they expand outward and increase in number. At the late stage, many micro-cracks overlap and penetrate. The crack number and range of the 60° specimen are smaller than those of the 30° specimen. This indicates that within the small-angle range, increasing the angle still weakens damage. Under medium-angle conditions, vertical propagation is suppressed. Horizontal propagation is dominant, with diagonal propagation as secondary. Damage is localized. Under large-angle conditions, micro-cracks initiate at the four corners of the fracture at the early stage. At the middle stage, they develop horizontally. At the late stage, a penetrating main crack forms on one side. Secondary cracks are extremely few. In general, the smaller the angle, the stronger the shear slip. Cracks develop progressively from multiple sources and the damage range is wider. The larger the angle, the more dominant the stress concentration. Cracks coalesce rapidly through a single path and the damage range is narrower. This determines the crack number and affects the load and deformation performance.

In summary, the fracture angle has a systematic regulating effect on the mechanical performance and damage evolution of single-intersecting fracture concrete. With a small angle, shear slip is dominant. Cracks develop progressively from multiple sources. The peak stress is high. The ultimate displacement is large. The crack number is large. Ductility is obvious. With a large angle, stress concentration is dominant. Cracks coalesce rapidly through a single path. All mechanical indicators deteriorate. Brittle features are obvious. The 30° specimen is significantly superior to the 150° specimen in peak stress (about twice), peak load (about 1 to 2 times), and final crack number (about three times). The qualitative trends and quantitative differences in these four dimensions are highly consistent. This reveals the regulating mechanism of fracture spatial configuration on concrete mechanical performance.

It should be distinguished that the angle obtained from the PFC2D numerical simulation in this paper is the intersection inner angle of fracture traces, which refers to the geometric included angle between two fracture traces in a two-dimensional plane. This trace angle is not equivalent to the fracture-plane dip angle in real three-dimensional engineering conditions. The latter characterizes the spatial geometric relationship between the normal direction of a spatial fracture plane and the axial loading direction. Two-dimensional simulations can only reflect the combined effect of in-plane fracture traces, and cannot represent the spatial inclination, outcropping of fracture planes or stress deflection in three-dimensional space. Accordingly, the laws derived from trace angles cannot be directly equated to the effects of fracture-plane dip angles on concrete mechanical behavior under three-dimensional conditions. For real-world engineering components, identical intersection angles of fracture traces paired with different fracture-plane dip angles will alter the ratio of normal stress to shear stress acting on fracture planes. This further changes the triggering condition of shear slip and the magnitude of crack-tip stress concentration, and eventually leads to remarkable differences in strength and failure modes.

The dominant controlled variables in this study are the trace intersection angle and offset magnitude of intersecting fractures. Parametric analyses concerning initial crack length, fracture aperture and contact friction on fracture surfaces have not been performed. Nevertheless, these factors can significantly modulate the mechanical responses of pre-cracked concrete. An increase in initial crack length amplifies stress concentration at crack tips, which generally reduces the peak strength of specimens and alters crack-initiation positions. Fracture aperture governs the particle-contact state on fracture surfaces. A large aperture removes nearly all contact friction on fracture surfaces, reduces shear-slip resistance, and makes slip-dominated failure along fracture planes more prone to occur. The contact-friction coefficient of fracture surfaces directly governs the energy-dissipation capacity during shear-slip processes. Higher friction can restrain interfacial shear slip and modify the generation scale of secondary cracks. Further multi-factor parametric investigations are recommended to establish damage-evolution criteria coupled with multiple fracture geometric parameters.

### 5.2. Effect of Double-Intersecting Fractures with Different Offset Degrees on Crack Development

Based on the stress–strain curves, load–displacement curves, and crack number evolution curves, the offset degree has a significant effect on the mechanical response of double-intersecting fracture concrete specimens. From the load-bearing characteristics, both peak stress and peak load show a grouping feature of first increasing and then stabilizing with increasing offset. The Zero-offset and Minor-offset specimens have relatively low and similar peak strengths. The Moderate-offset, Substantial-offset, and Maximum-offset specimens have relatively high and similar peak strengths. The reason is that under low-offset conditions, the two fracture groups are spatially close and have an obvious coalescence trend. During loading, a single stress-concentration zone is easily formed. This causes particle bonds to fail in a concentrated manner at a lower load level. Increasing the offset disrupts the continuous weak plane. Stress distribution becomes more dispersed. The load capacity improves. However, after the offset exceeds the Moderate level, this optimization effect tends to saturate. The peak strength no longer increases significantly.

In terms of deformation, the patterns shown by the stress–strain curves and load–displacement curves are not completely consistent. The ultimate strain is largest for the Substantial-offset specimen and smallest for the Maximum-offset specimen. However, the final displacement generally increases with increasing offset. This indicates that the ultimate strain mainly reflects the uniform plastic deformation capacity before failure. The final displacement also includes the slip contribution after the formation of the main crack. Under low-offset conditions, damage is concentrated. Plastic deformation reserve is insufficient. The ultimate strain is relatively low. Under Moderate- and Substantial-offset conditions, the fracture offset delays bond breakage. The plastic deformation capacity is enhanced. Under the Maximum-offset condition, the diagonal coalescence path is clear. Crack propagation accelerates. The plastic stage is compressed. The ultimate strain decreases instead. However, the shear slip caused by the diagonal main crack continues to increase the loading point displacement. Therefore, the improved deformation capacity mainly comes from local slip rather than uniform plastic deformation. The risk of brittle failure is higher.

In terms of crack number, the final crack number increases with offset within a certain scope but exhibits non-monotonic characteristics. The Zero-offset specimen produces the fewest cracks, and the peak value of crack number is obtained under the Substantial-offset condition, followed by a moderate decrease for the Maximum-offset specimen. Under low-offset conditions, fracture coalescence is smooth. Crack propagation is relatively concentrated. Secondary cracks are limited. As the offset increases, the spatial overlapping relationship becomes more disordered. The number of stress-concentration zones increases. Secondary micro-cracks develop widely. The total crack number increases accordingly. At the same time, the offset degree changes the coalescence direction of the main crack. Zero-offset and Minor-offset specimens show mainly central coalescence. Moderate-offset specimens show vertical coalescence. Substantial-offset specimens show diagonal coalescence from upper-left to lower-right. This indicates that the fracture offset regulates both the damage scale and the failure-surface morphology by changing the internal stress path. In addition, the final crack number of double-intersecting fracture specimens is generally lower than that of single-intersecting fracture specimens. Damage tends to be more concentrated rather than fully dispersed. This explains from a meso-perspective why their mechanical performance is weaker.

### 5.3. Research Prospects

This paper adopts the two-dimensional discrete element PFC2D simulation method to reveal how the intersection angle of single pre-fabricated intersecting fractures and offset level of double pre-fabricated intersecting fractures govern the macroscopic mechanical responses and mesoscopic crack propagation behaviors of concrete under uniaxial compression, clarifying the competing mechanism between shear-slip effect and stress-concentration effect in defective concrete. Nevertheless, the present work still has several evident limitations: the mesoscopic parameters are selected from published literature without targeted laboratory calibration, so absolute quantitative simulation outputs cannot be directly applied in practical engineering [41,42]; the 2-D plane model fails to reproduce real three-dimensional fracture geometry and spatial stress state; only uniaxial compressive loading is considered, ignoring the combined influence of fracture length, fracture surface friction, seepage and in situ stress. In addition, repeated trial-and-error attempts are required for DEM parameter selection, which brings heavy computational cost to multi-condition parametric analysis. In follow-up studies, laboratory test data shall be combined to calibrate mesoscopic parameters, three-dimensional discrete element models shall be constructed, and multi-field coupling loading conditions shall be further considered. Meanwhile, artificial-intelligence and machine-learning techniques [43,44,45] can be introduced: large datasets generated by massive DEM numerical simulations can be used to train surrogate models to realize intelligent and fast inversion of DEM mesoscopic parameters, establish mapping relationships between fracture geometric parameters and concrete failure characteristics, accelerate multi-factor parametric screening, and provide an efficient data-driven tool for safety evaluation and reinforcement design of fractured concrete support structures in underground mines. In addition, only uniaxial compressive loading was considered in the current parametric simulation. Although intersecting-fracture damage under compression is highly relevant for the safety evaluation of sidewall shotcrete supports, underground shotcrete linings (e.g., mine roof structures) are frequently subjected to bending-dominated or bending–shear combined stress states. Even so, local compressive zones still exist in bending-loaded components. The mesoscopic crack-interaction mechanism revealed in this work can provide baseline references for future investigations concerning fractured concrete under bending or complex mixed-stress conditions. Further numerical or experimental work is required to reproduce such multi-stress-coupled scenarios.

## 6. Conclusions

This paper uses the PFC discrete element simulation method. It systematically studies the influence of single fractures with different angles and double fractures with different offsets on crack development in concrete under uniaxial compression. The main conclusions are as follows:

(1) The angle of a single-intersecting fracture has a systematic effect on the mechanical performance of the specimen. A smaller angle allows more sufficient shear slip. Cracks develop progressively from multiple sources. Stress distribution is relatively uniform. The peak stress, peak load, and ultimate strain are higher. The final crack number (sum of tensile and shear micro-cracks) is also larger. A larger angle suppresses shear slip. Stress concentration at the fracture tip intensifies. Cracks propagate rapidly through a single coalescence path. The load capacity and deformation capacity both decrease. Brittle failure features become obvious.
(2) The effect of offset degree on the load capacity of double-intersecting fractures shows a grouping feature of first increasing and then stabilizing. The Zero-offset and Minor-offset specimens have relatively low and similar peak strengths. The Moderate-offset, Substantial-offset, and Maximum-offset specimens have relatively high and similar peak strengths. The mechanism is that under low offset, fracture coalescence is smooth. A single stress-concentration zone is easily formed. Increasing the offset causes fracture dislocation. This breaks the continuous weak plane. Stress distribution becomes more dispersed. The load capacity improves. However, after the offset exceeds the moderate level, the optimization effect tends to saturate.
(3) The deformation response of double-intersecting fractures shows an inconsistency between ultimate strain and final displacement. The ultimate strain is largest for the Substantial-offset specimen and smallest for the Maximum-offset specimen. However, the final displacement generally increases with increasing offset. This indicates that the improved deformation capacity under the Maximum-offset condition mainly comes from shear slip after the diagonal main crack forms, rather than from uniform plastic deformation before failure. The risk of brittle failure is actually higher.
(4) For specimens with double pre-fabricated intersecting fractures, the final crack number increases with offset within a certain range instead of showing strict monotonic growth. The Zero-offset specimen generates the fewest cracks, while the maximum crack number occurs under the Substantial-offset condition. Further increasing offset to the Maximum-offset level will reduce the total crack quantity. Meanwhile, ultimate strain does not increase monotonically with offset: the Substantial-offset case possesses the largest ultimate strain, while the Maximum-offset case presents the smallest ultimate strain, despite its relatively large final displacement contributed by post-peak shear slip. As offset increases, the spatial overlapping relationship of fractures becomes more disordered, and more stress-concentration regions emerge to trigger abundant secondary micro-cracks. The coalescence direction of the main crack changes successively from central-horizontal, to vertical, and then to diagonal. It demonstrates that offset regulates both damage scale and failure-surface morphology by altering internal stress transfer paths.
(5) Comparing single and double-intersecting fractures, the final crack number of double-intersecting fracture specimens is generally lower than that of single-intersecting fracture specimens. Damage tends to be more concentrated rather than fully dispersed. The macroscopic mechanical performance is also weakened accordingly. The regulation of concrete mechanical performance by fracture spatial configuration is essentially the result of competition between two mechanisms: shear slip and stress concentration. The damage distribution characteristic, rather than the total crack number, determines the degree of macroscopic deterioration. In actual engineering inspection, special attention should be paid to large-angle single-intersecting fractures, Zero-offset or Minor-offset double-intersecting fractures, and the diagonal coalescence paths of Maximum-offset double-intersecting fractures. These locations are prone to become hidden dangers for sudden failure.

It should be noted that the above understandings are obtained from two-dimensional PFC2D parametric simulations without experimental calibration. The absolute mechanical indices derived from this idealized numerical model cannot be directly adopted for quantitative safety assessment, reinforcement design or field inspection of real concrete structures. Several inherent limitations should be highlighted: mesoscopic parameters are mainly derived from published literature rather than matching tests for the material in situ; the 2-D plane assumption cannot reproduce the true three-dimensional stress state and spatial fracture morphology of engineering concrete; loading and boundary conditions are simplified to uniaxial compression. When transferring these findings to practical projects, further targeted laboratory tests and three-dimensional numerical investigation are necessary to eliminate model-induced deviations. Existing two-dimensional simulations cannot capture out-of-plane crack propagation and spatial interpenetration of fractures, which commonly occur in real-world concrete components. Such planar simplification may underestimate certain complex failure modes driven by three-dimensional stress. Therefore, extra caution is required when extrapolating the simulated crack-coalescence patterns to actual engineering practice.

## Figures and Tables

**Figure 1:**
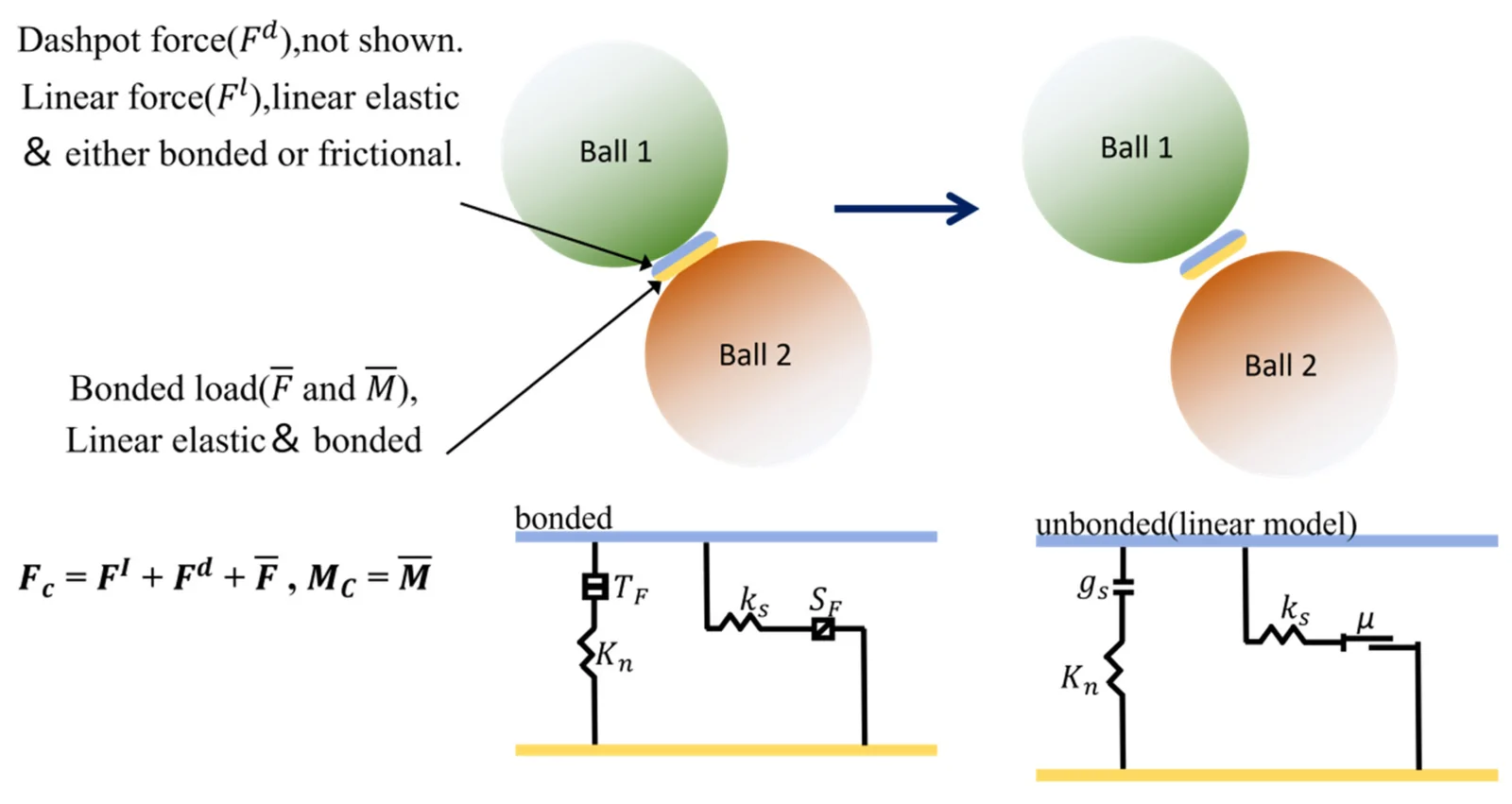
PFC parallel-bond model.

**Figure 2:**
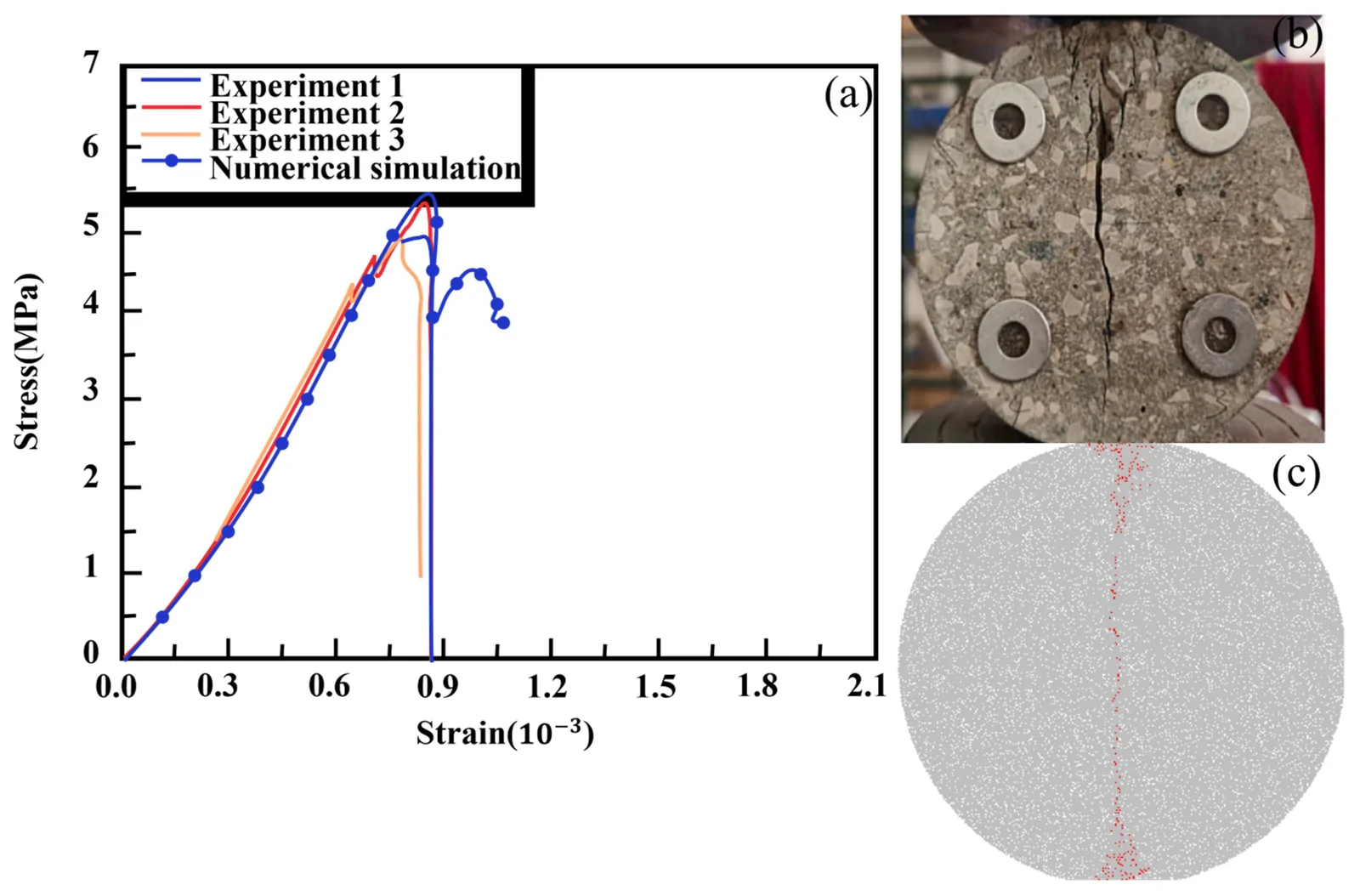
Calibration results of mesoscopic parameters. Quoted from the paper by Ren et al. [40] (**a**) Comparison of stress–strain curves between PFC2D numerical simulation and three groups of experimental results for intact concrete specimens; (**b**) typical failure morphology of intact concrete specimen under uniaxial compression; (**c**) the simulation results of crack propagation in PFC.

**Figure 3:**
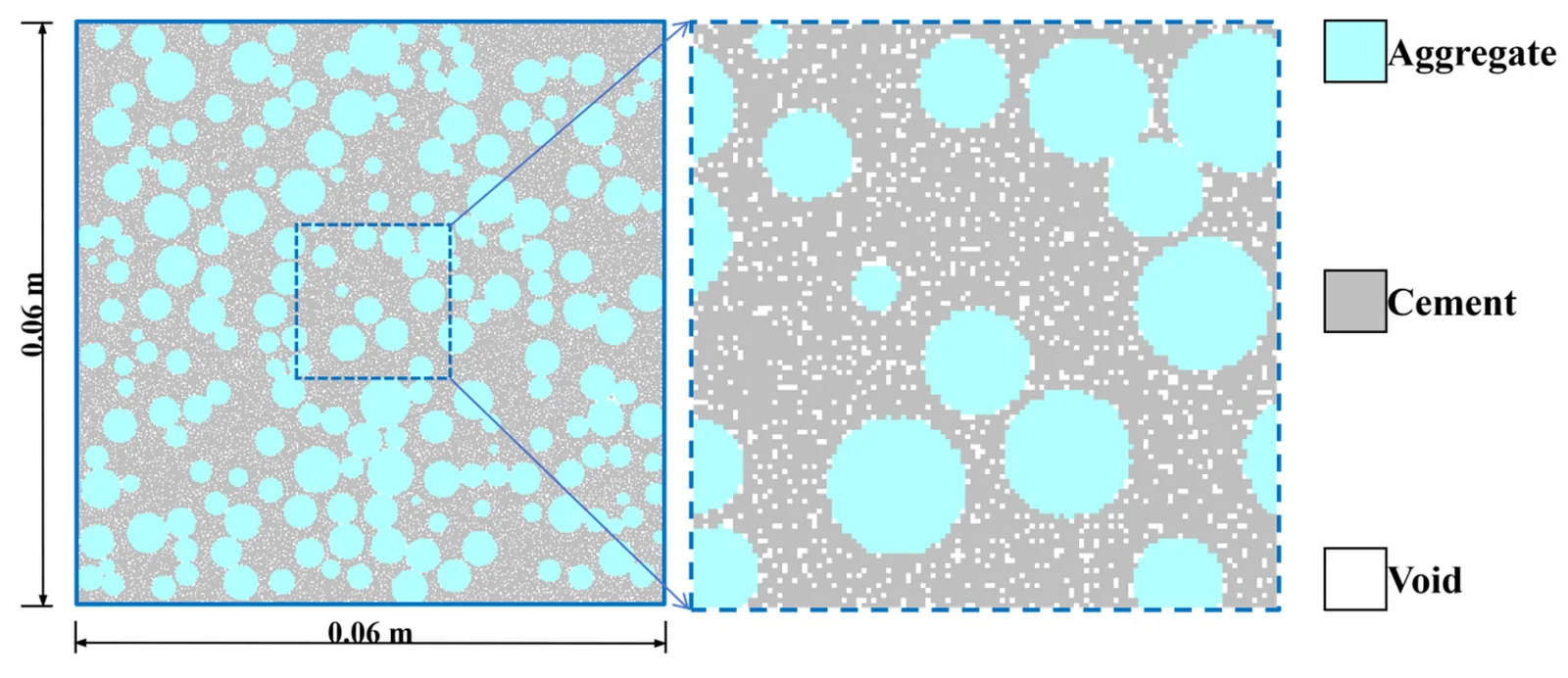
Mesoscale model of concrete.

**Figure 4:**
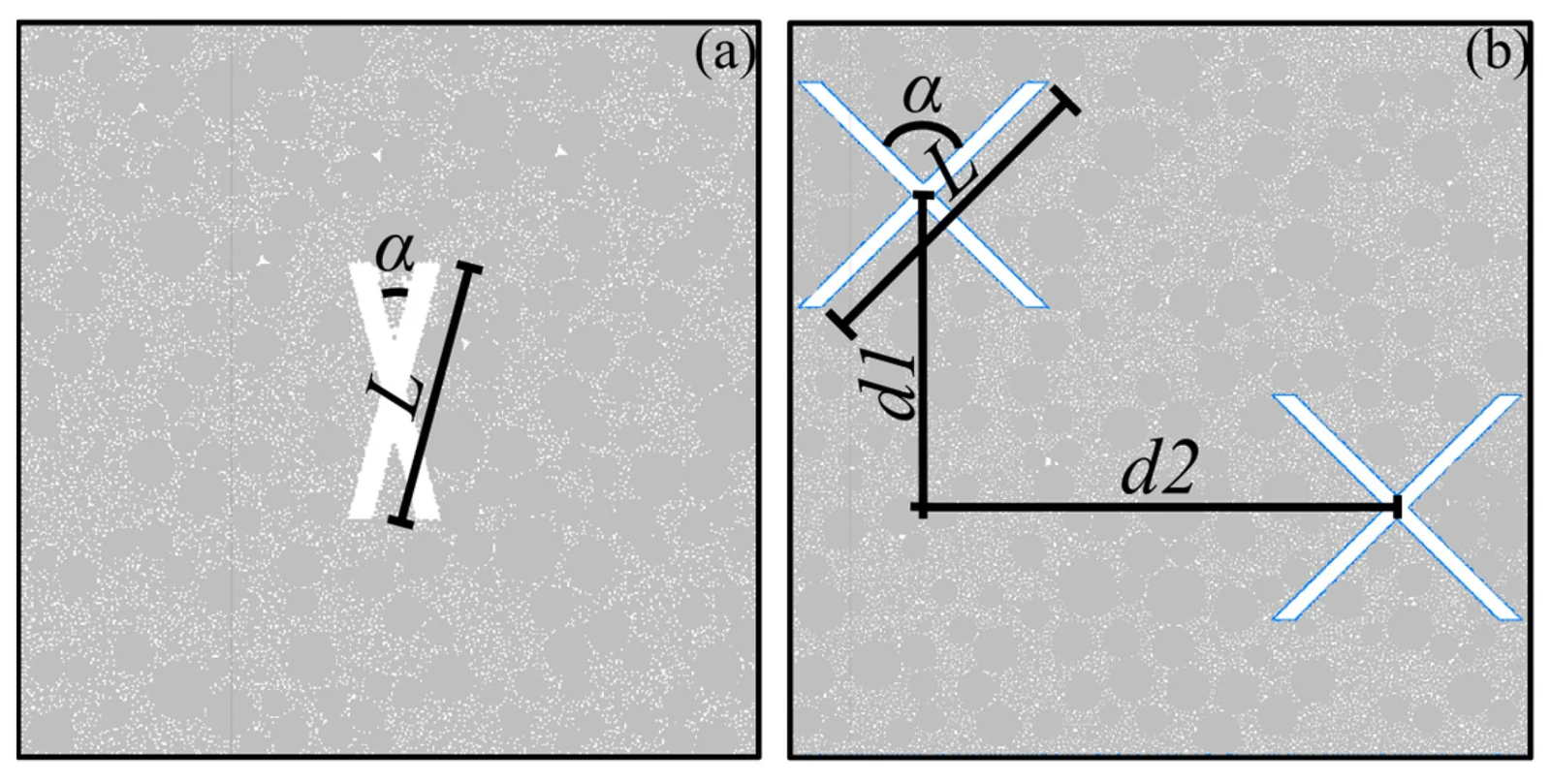
Schematic diagram of pre-fabricated intersecting fracture defects in PFC2D numerical specimens. (**a**) Configuration of single-intersecting fracture, marking the upper-side internal included angle *α*, constant fracture trace length *L*. (**b**) Configuration of double offset intersecting fractures. *α* is the fracture intersection angle, *L* denotes fixed fracture trace length for all cases; *d*1 represents the vertical offset distance and *d*2 represents the horizontal offset distance between two sets of intersecting fractures.

**Figure 5:**
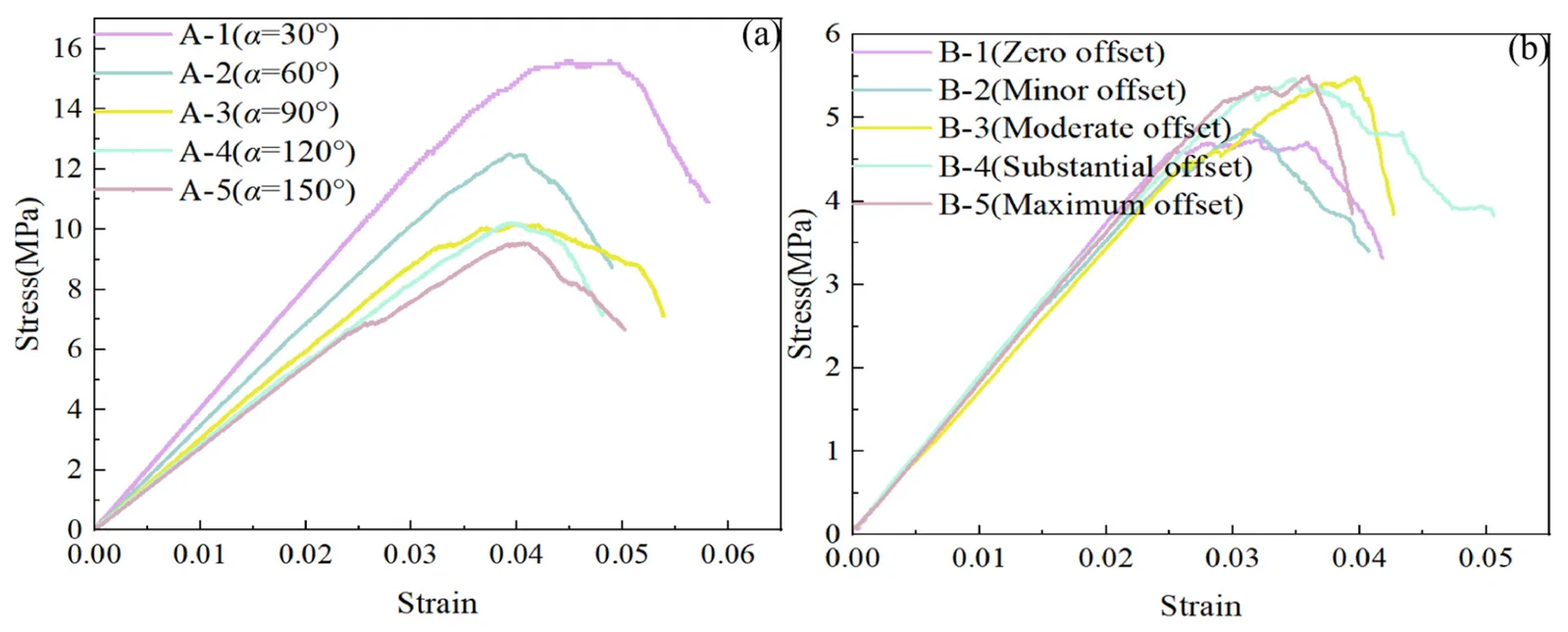
Stress–strain curves for specimens under different conditions: (**a**) The single-intersecting fracture has different angles. (**b**) The double-intersecting fracture has different offsets.

**Figure 6:**
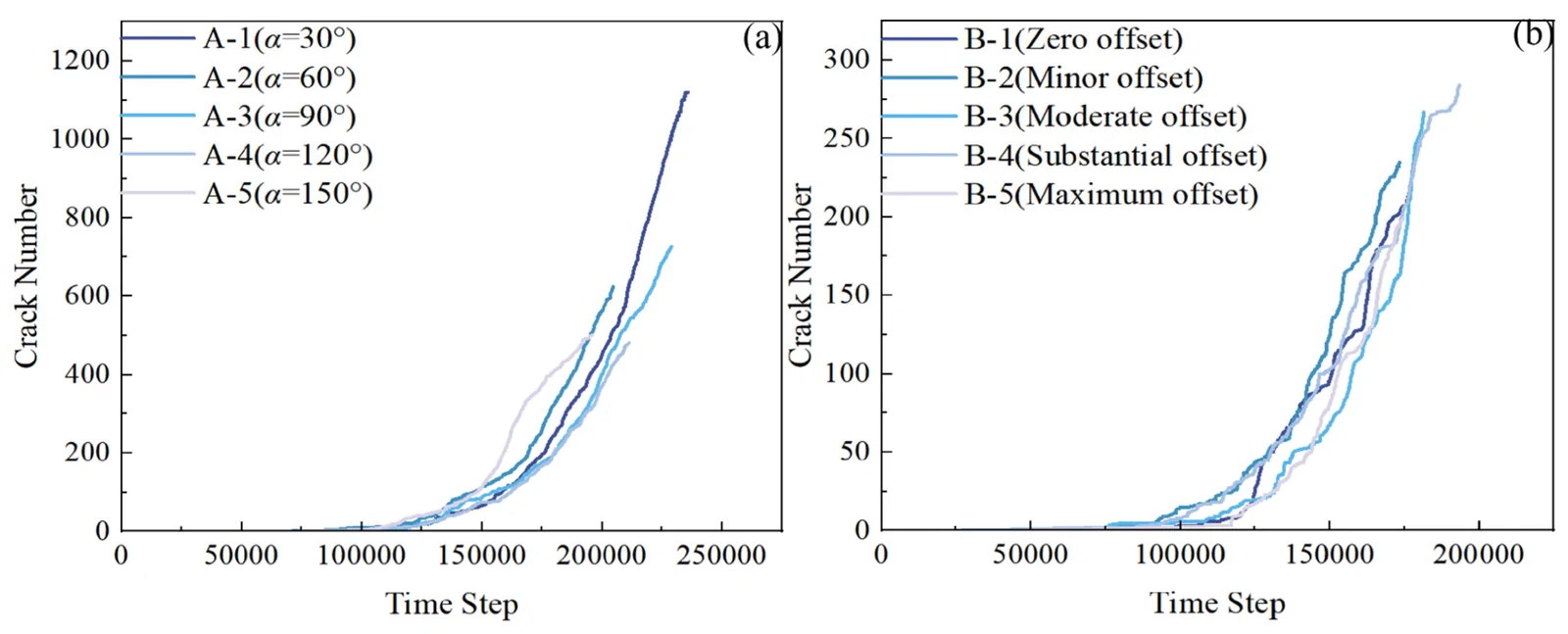
Crack number evolution curves for specimens under different conditions: (**a**) The single-intersecting fracture has different angles. (**b**) The double-intersecting fracture has different offsets.

**Figure 7:**
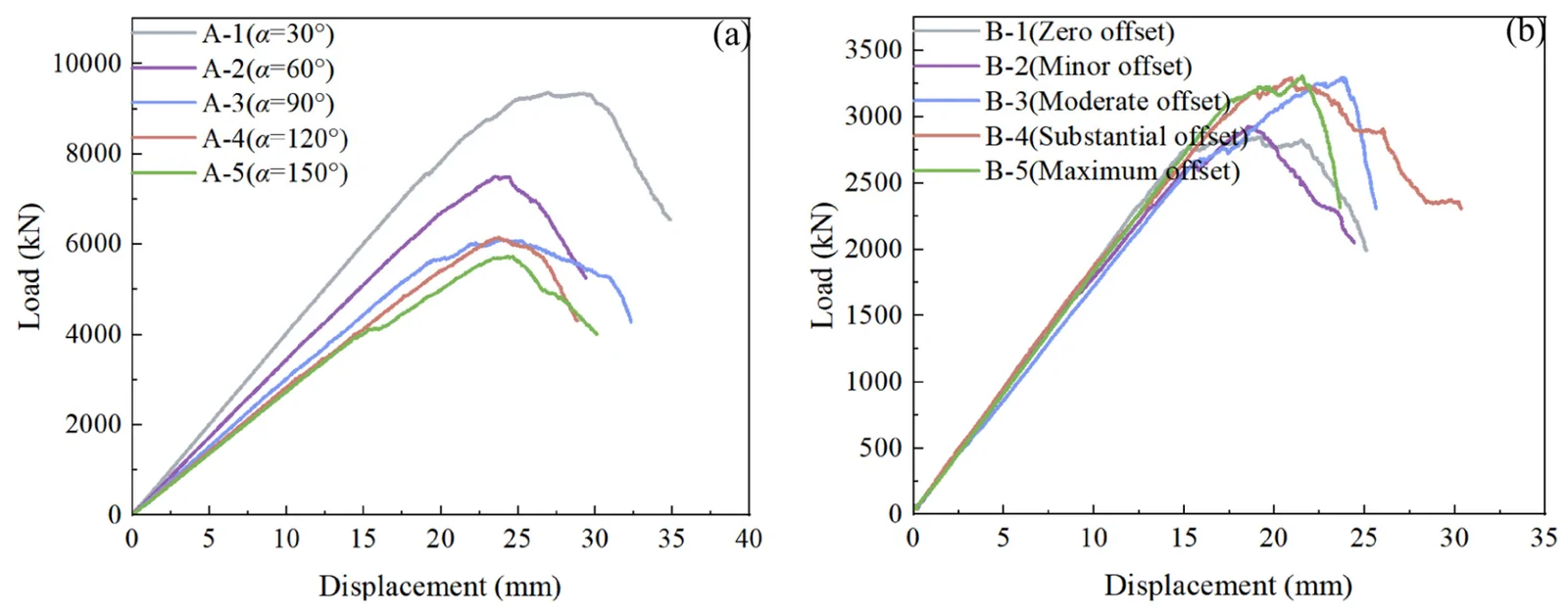
Load–displacement curves for specimens under different conditions: (**a**) the single-intersecting fracture has different angles. (**b**) The double-intersecting fracture has different offsets.

**Figure 8:**
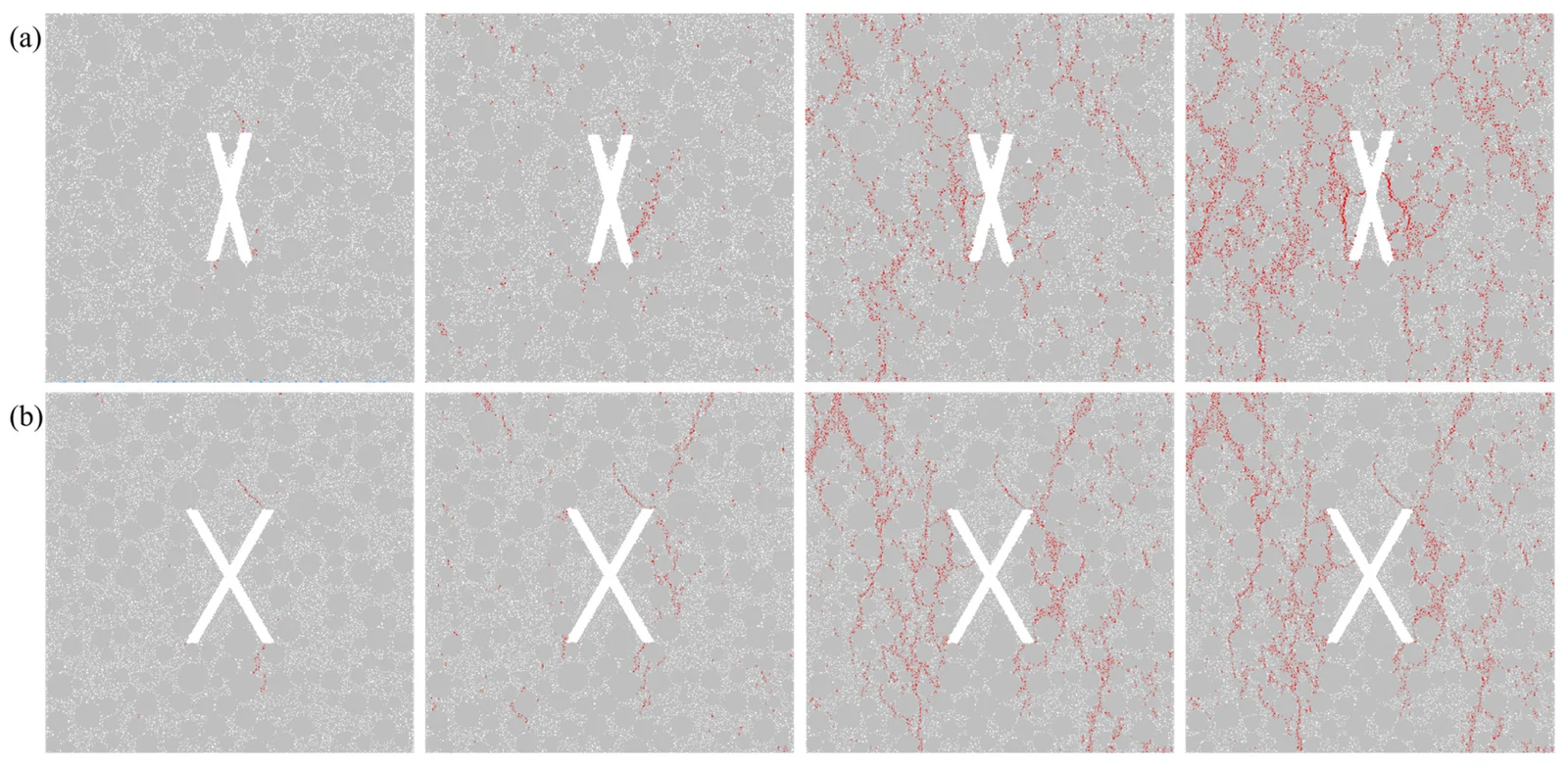
Crack propagation snapshots of specimens with pre-fabricated single-intersecting fractures. Subplots from left to right represent the early-loading stage (crack initiation), mid-loading stage (crack growth), and two successive late-loading stages approaching unstable failure. (**a**) Fracture angle of 30°; (**b**) fracture angle of 60°.

**Figure 9:**
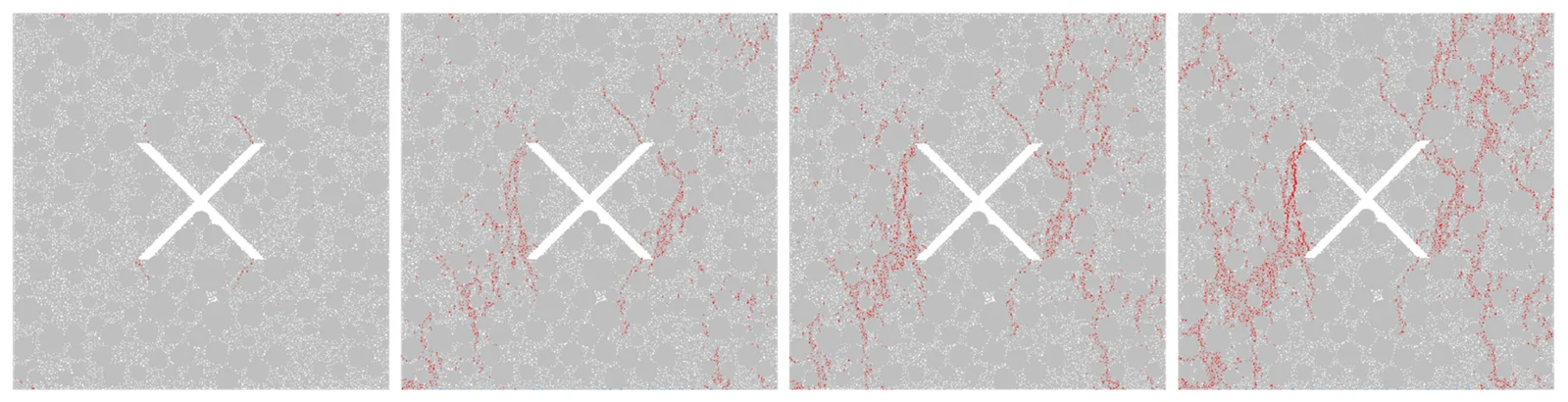
Crack propagation snapshots of the specimen with a pre-fabricated single-intersecting fracture (fracture angle of 90°). Subplots from left to right represent the early-loading stage (crack initiation), mid-loading stage (crack growth), and two successive late-loading stages approaching unstable failure.

**Figure 10:**
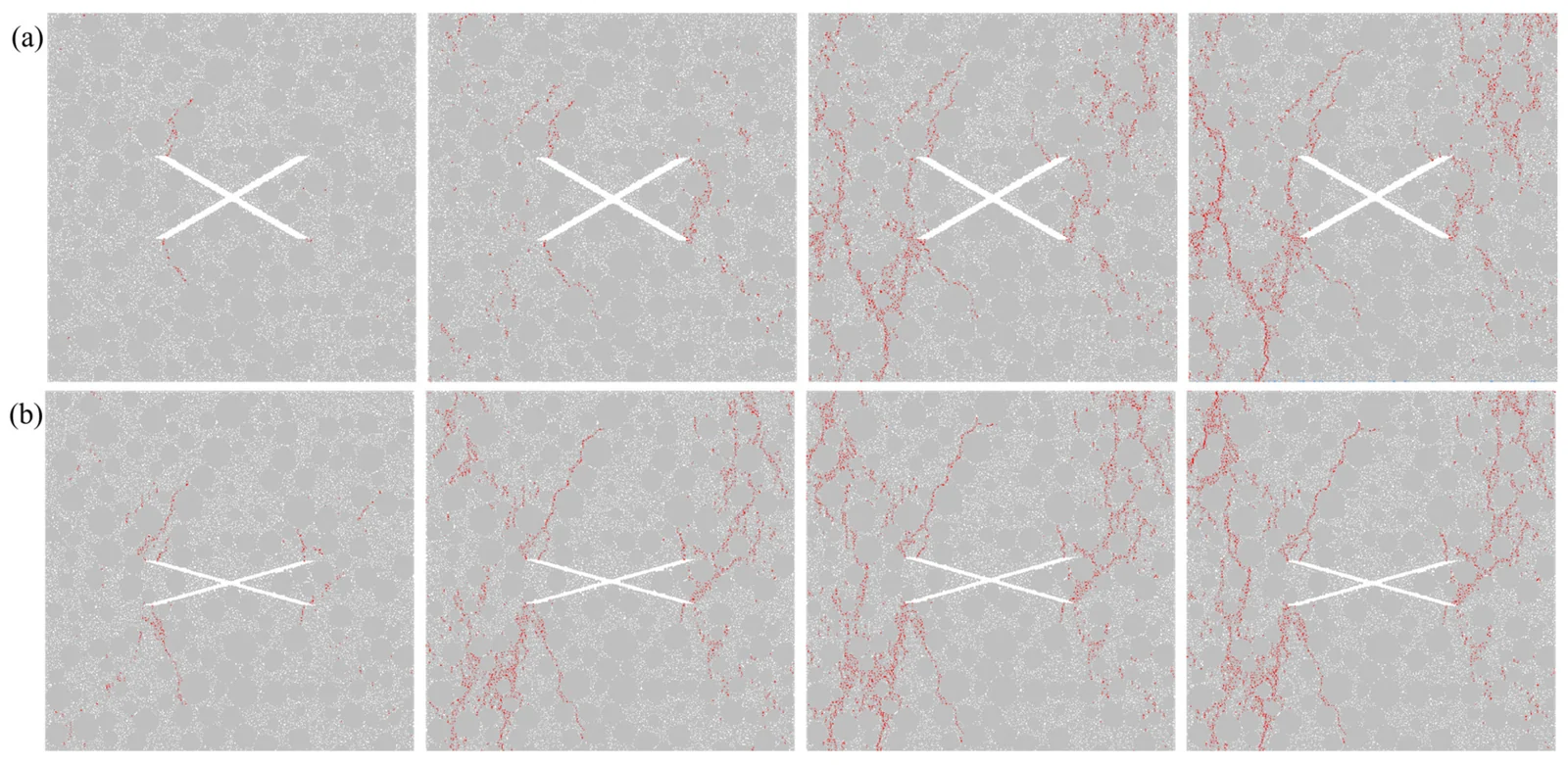
Crack propagation snapshots of specimens with pre-fabricated single-intersecting fractures. Subplots from left to right represent the early-loading stage (crack initiation), mid-loading stage (crack growth), and two successive late-loading stages approaching unstable failure. (**a**) Fracture angle of 120°; (**b**) fracture angle of 150°.

**Figure 11:**
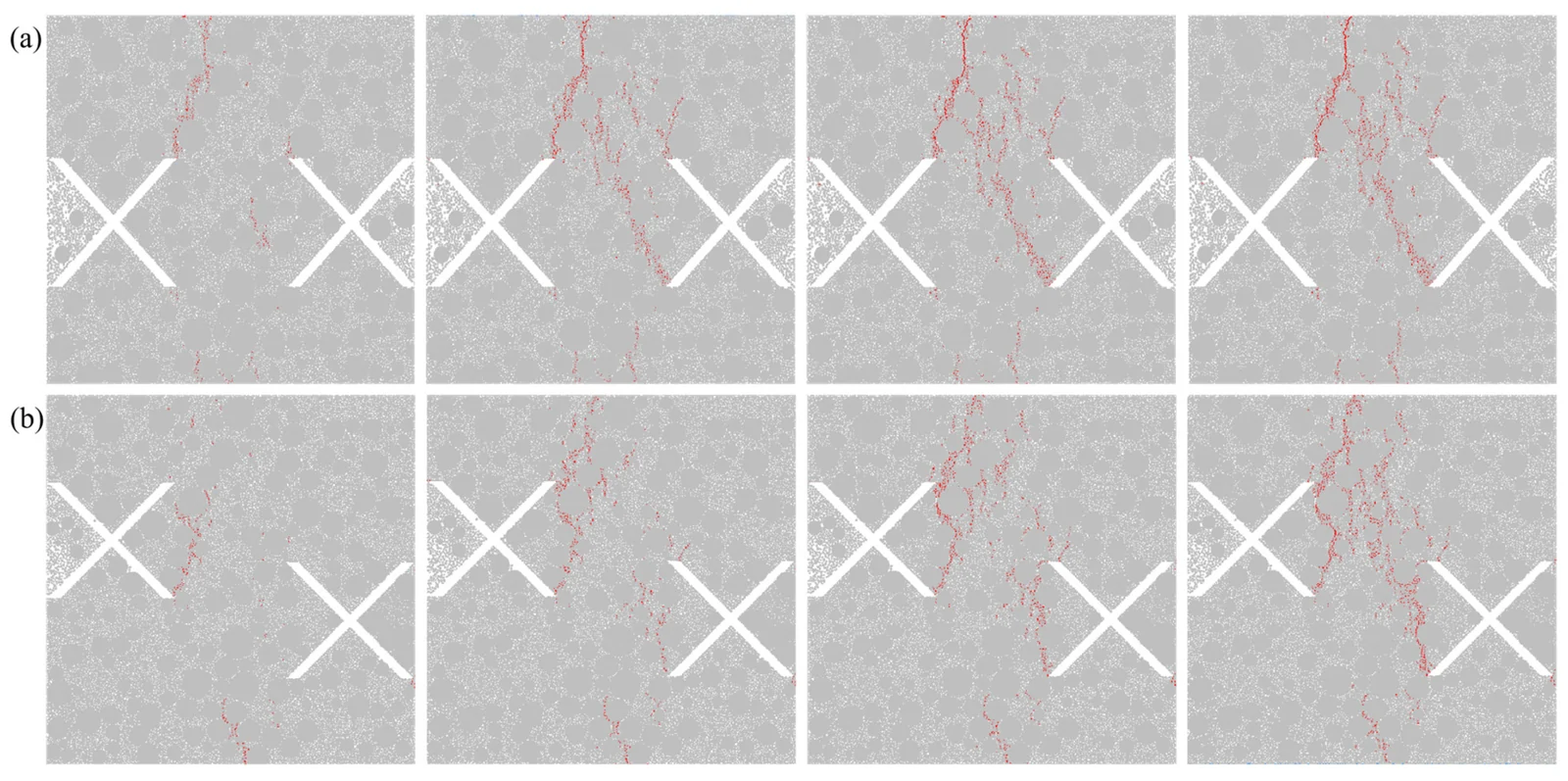
Crack propagation snapshots of specimens with pre-fabricated double-intersecting fractures. Subplots from left to right represent the early-loading stage (crack initiation), mid-loading stage (crack growth), and two successive late-loading stages approaching unstable failure. (**a**) Zero-offset condition; (**b**) Minor-offset condition.

**Figure 12:**
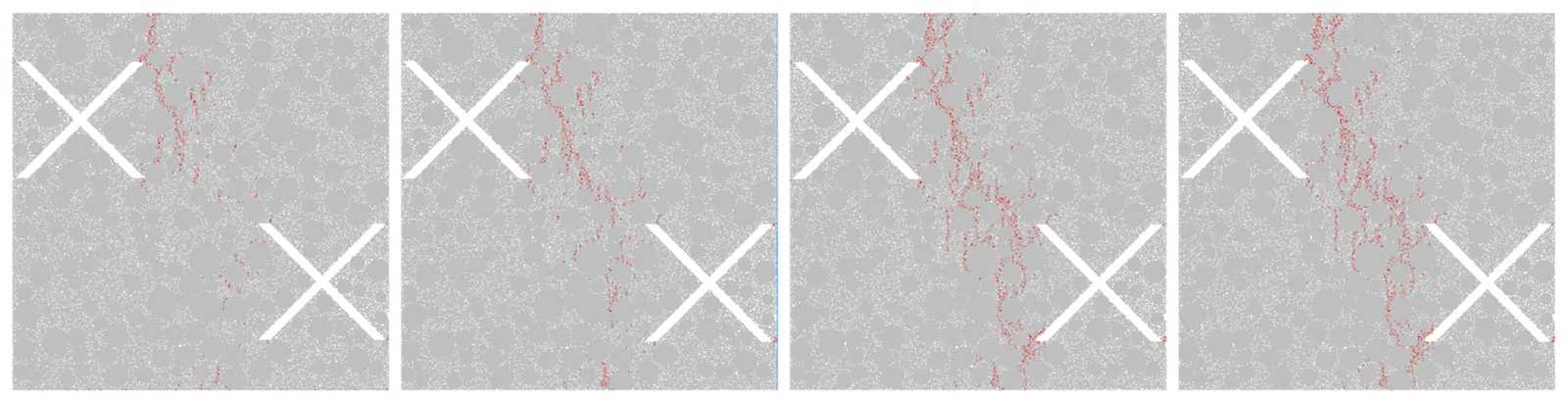
Crack propagation snapshots of the specimen with pre-fabricated double-intersecting fractures under the Moderate-offset condition. Subplots from left to right represent the early-loading stage (crack initiation), mid-loading stage (crack growth), and two successive late-loading stages approaching unstable failure.

**Figure 13:**
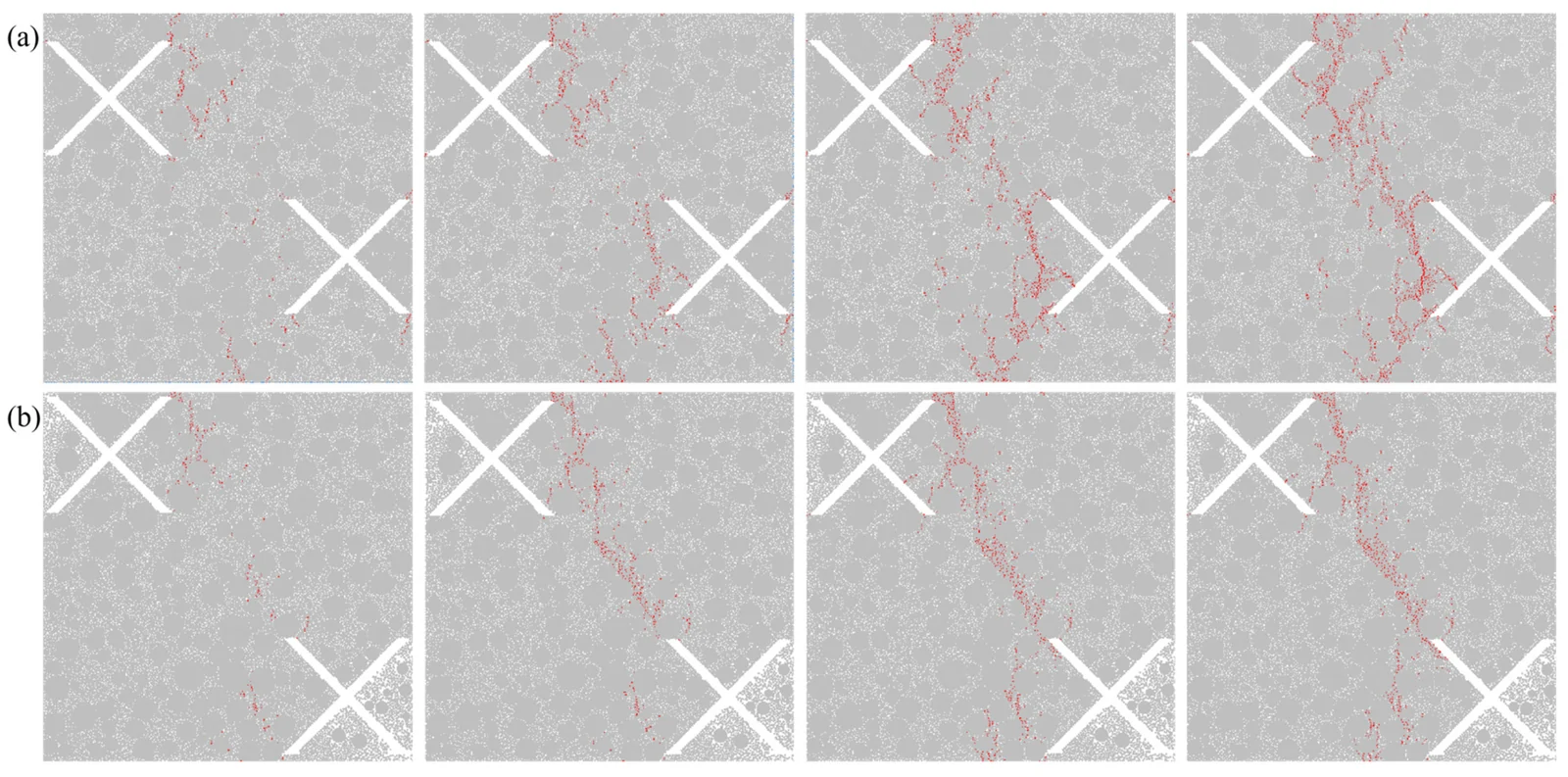
Crack propagation snapshots of specimens with pre-fabricated double-intersecting fractures. Subplots from left to right represent the early-loading stage (crack initiation), mid-loading stage (crack growth), and two successive late-loading stages approaching unstable failure. (**a**) Substantial-offset condition; (**b**) Maximum-offset condition.

**Table 1:** Mesoscopic parameters of the PFC model [39].

Parameters of Mortar		Parameters of Aggregates	
Emod (Pa)	$130\;\times \;10^{8}$	Emod (Pa)	$600\;\times \;10^{8}$
Pb_emod (Pa)	$130\;\times \;10^{8}$	Pb_emod (Pa)	$600\;\times \;10^{8}$
Pb_ten (Pa)	$7\;\times \;10^{6}$	Pb_ten (Pa)	$20\;\times \;10^{6}$
Pb_coh (Pa)	$8\;\times \;10^{6}$	Pb_coh (Pa)	$25\;\times \;10^{6}$
Pb_PFA (°)	45	Pb_PFA (°)	40
Kratio	2	Kratio	1.5
damp	0.7	damp	0.7

**Table 2:** Summary of mechanical and crack-related indices under different working conditions.

Specimen ID	Peak Stress (MPa)	Ultimate Strain	Peak Load (kN)	Final Crack Number
A-1 (α = 30°)	15.6	0.058	9363.46	235,740
A-2 (α = 60°)	12.5	0.049	7501.46	204,650
A-3 (α = 90°)	10.2	0.054	6109.16	228,980
A-4 (α = 120°)	10.2	0.048	6148.77	211,170
A-5 (α = 150°)	9.5	0.050	5729.65	195,870
B-1 (Zero offset)	4.7	0.042	2845.05	177,340
B-2 (Minor offset)	4.9	0.041	2924.73	173,410
B-3 (Moderate offset)	5.5	0.043	3295.88	181,520
B-4 (Substantial offset)	5.5	0.051	3291.56	193,510
B-5 (Maximum offset)	5.5	0.039	3306.15	174,530

**Note:** The final crack number is the sum of tensile micro-cracks and shear micro-cracks caused by parallel-bond failure at particle contacts. Macro-cracks formed by micro-crack coalescence are not counted separately.

## Data Availability

The original contributions presented in this study are included in the article. Further inquiries can be directed to the corresponding author.
